# A Novel Platelet-Related Gene Signature for Predicting the Prognosis of Triple-Negative Breast Cancer

**DOI:** 10.3389/fcell.2021.795600

**Published:** 2022-01-12

**Authors:** Jindong Xie, Yutian Zou, Feng Ye, Wanzhen Zhao, Xinhua Xie, Xueqi Ou, Xiaoming Xie, Weidong Wei

**Affiliations:** ^1^ Department of Breast Oncology, State Key Laboratory of Oncology in South China, Sun Yat-sen University Cancer Center, Collaborative Innovation Center for Cancer Medicine, Guangzhou, China; ^2^ Department of Radiotherapy, The First Affiliated Hospital, Hengyang Medical School, University of South China, Hengyang, China

**Keywords:** triple-negative breast cancer, platelet, mRNA signature, prediction, prognosis

## Abstract

Regarded as the most invasive subtype, triple-negative breast cancer (TNBC) lacks the expression of estrogen receptors (ERs), progesterone receptors (PRs), and human epidermal growth factor receptor 2 (HER2) proteins. Platelets have recently been shown to be associated with metastasis of malignant tumors. Nevertheless, the status of platelet-related genes in TNBC and their correlation with patient prognosis remain unknown. In this study, the expression and variation levels of platelet-related genes were identified and patients with TNBC were divided into three subtypes. We collected cohorts from The Cancer Genome Atlas (TCGA) and the Gene Expression Omnibus (GEO) databases. By applying the least absolute shrinkage and selection operator (LASSO) Cox regression method, we constructed a seven-gene signature which classified the two cohorts of patients with TNBC into low- or high-risk groups. Patients in the high-risk group were more likely to have lower survival rates than those in the low-risk group. The risk score, incorporated with the clinical features, was confirmed as an independent factor for predicting the overall survival (OS) time. Functional enrichment analyses revealed the involvement of a variety of vital biological processes and classical cancer-related pathways that could be important to the ultimate prognosis of TNBC. We then built a nomogram that performed well. Moreover, we tested the model in other cohorts and obtained positive outcomes. In conclusion, platelet-related genes were closely related to TNBC, and this novel signature could serve as a tool for the assessment of clinical prognosis.

## Introduction

Breast cancer (BC) remains a primary disease burden for women worldwide (Britt et al., 2020). According to the expression of estrogen receptors (ERs), progesterone receptors (PRs), and human epidermal growth factor receptor 2 (HER2) proteins, BC can be divided into several subtypes, and each lead to certain therapeutic sensitivities and prognoses (Heer et al., 2020). Among these subtypes, triple-negative breast cancer (TNBC) accounts for 15–20% of all breast cancer cases (Carey et al., 2010). Fewer than 30% of metastatic TNBC patients survive for more than 5 years (Johnson et al., 2013). Due to its chemoresistance and unfavorable prognosis, treatment of TNBC is still a major challenge and considered to be a “black hole” compared to other BC subtypes (Zheng et al., 2020a). Given the limitations of TNBC treatments, there is an urgent need to explore novel targets to ameliorate the prognosis of TNBC, and effective models are imperative to make targeted therapy more feasible.

Platelets, which regulate hemostasis and thrombosis, are one of the three main types of blood cells in the human body (Holinstat, 2017). They are created in the bone marrow and circulate through the bloodstream. When bleeding or injury occurs, they quickly reach the wound site, form a plug, and recruit more platelets simultaneously. They form a clot that coalesces with other clotting factors to help stop bleeding (van der Meijden and Heemskerk, 2019). A reduction of platelets can result in bleeding diathesis, causing spot bleeding, bruising, and purple spots. When the platelet count lowers even more, hematencephalon or pneumorrhagia can occur, which lead to death (Vinholt, 2019). In addition to playing an indispensable role in clotting and maintaining hemostasis, platelets are also directly implicated in cancer. It is well known that platelets participate substantially in the growth and metastasis of cancers. In 1872, Leopold Riess found that thrombocytosis was common in solid tumors (Riess, 1872). In many patients with cancer, platelet counts can greatly increase (Haemmerle et al., 2018). Platelets are involved in angiogenesis and tumor progression. It has been reported that thrombocytosis is directly associated with a shorter survival time in various types of cancer (Buergy et al., 2012). Platelets have an impact on the treatment efficacy of patients and take part in multiple steps of cancer metastasis. Tumor cells cannot survive unless they avoid attack by natural killer (NK) cells. Platelets encase the tumor cells in a thrombus so that they escape from NK cell monitoring, which allows them to circulate through the bloodstream (Nieswandt et al., 1999). Additionally, platelets can secrete TGF-β, which enhances metastasis and reduces the cytotoxicity of NK cells and the production of IFN-γ (Kopp et al., 2009). Platelets can also store and release growth factors such as the vascular endothelial growth factor (VEGF) and platelet-derived growth factor (PDGF), which are crucial for tumor growth and vascular stability, when stimulated by external sources (Wojtukiewicz et al., 2017). Moreover, platelets can prevent chemotherapy-induced apoptosis in cancer cells. It has been proven that thrombocytosis is closely related to poor response to chemotherapy during *in vitro* and *in vivo* experiments, which indicates that the efficacy of chemotherapy drugs could be improved if we suppress the amount or activity of platelets (Bottsford-Miller et al., 2015).

**FIGURE 1:**
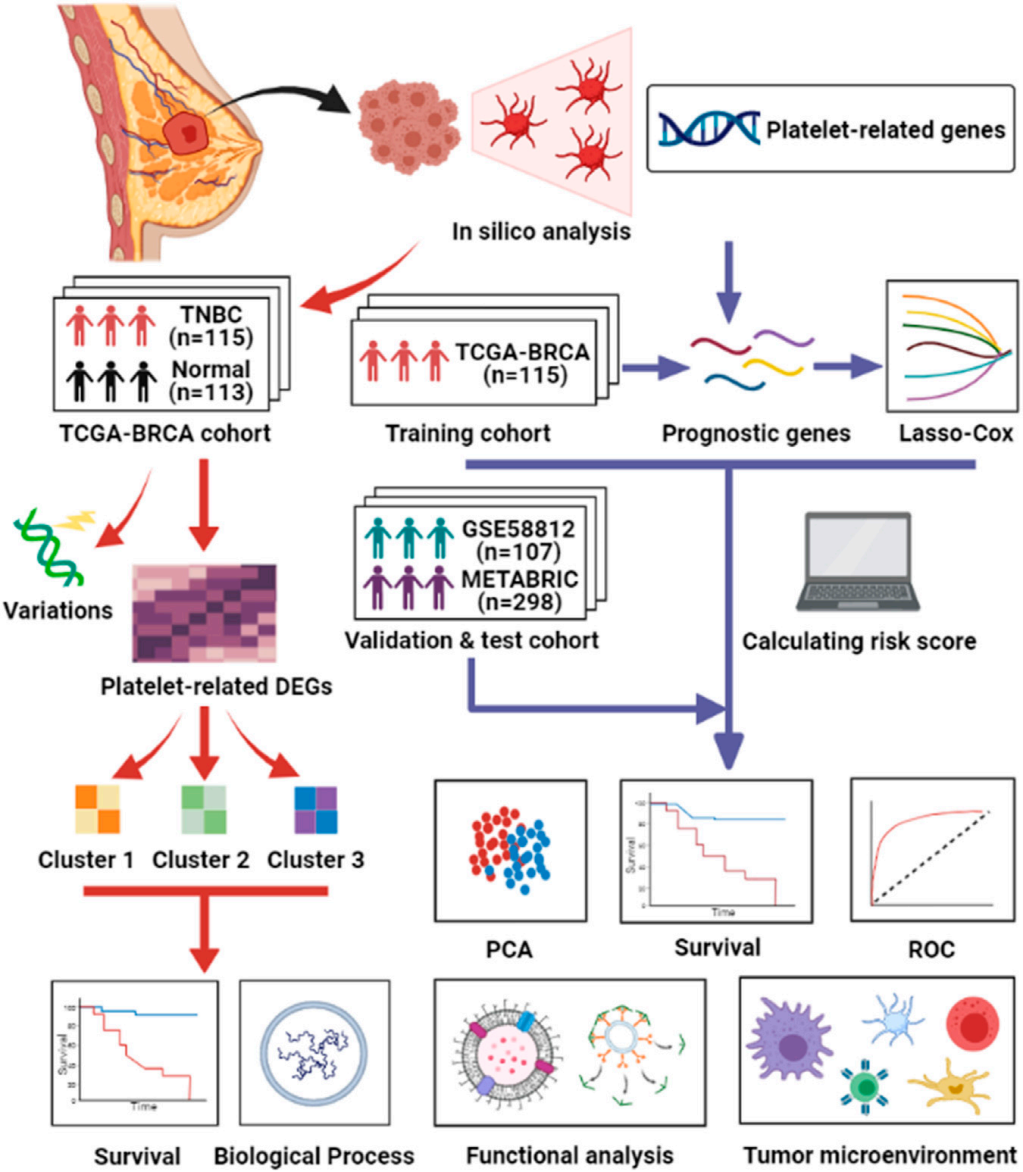
Workflow of data analysis in our study.

Based on the existing findings above, we hypothesized that platelets are closely related to the proliferation and metastasis of cancers. However, there are few studies on the detailed functions of platelets in TNBC. In addition, array-based databases to recognize survival-associated genes are valuable and urgently needed to guide tailored therapies for TNBC patients (Xie et al., 2019). Therefore, we conducted a comprehensive study to examine the status of platelet-related genes in normal breast and TNBC tissues, determine their prognostic value, and determine the relationship between platelets and the tumor immune microenvironment. Figure 1 summarizes the workflow of the data analysis. In summary, our study could contribute to discovering the heterogeneity of TNBC and offers a method to select suitable patients for immunotherapy.

## Materials and Methods

### Selection of Platelet-Related Genes

The platelet-related genes list was collected from gene set enrichment analysis (GSEA) gene sets (https://www.gsea-msigdb.org/gsea/index.jsp/) by the keyword “platelet.” Finally, 480 genes related to platelets were included in the analysis (Supplementary Table S1).

### Data Collection

We acquired the raw transcriptome count data and the normalized converted RNA-sequencing (RNA-seq) profile fragments per kilobase of exon per million reads mapped (FPKM) of 115 TNBC patients and 113 normal tissues from the University of California Santa Cruz (UCSC) database. Clinical characteristics and copy number variation (CNV) information were also downloaded (https://xenabrowser.net/datapages/). Masked somatic mutation information was downloaded from the GDC Data Portal (https://portal.gdc.cancer.gov/). The log2-converted chip-seq data and clinical information of the external 107 TNBC patients’ validation cohort were obtained from the Gene Expression Omnibus (GEO) database (ID: GSE58812). We also collected DNA microarray data and clinical information of 298 TNBC patients from the Molecular Taxonomy of Breast Cancer International Consortium (METABRIC) dataset as a test cohort (http://molonc.bccrc.ca/aparicio-lab/research/metabric/). Another dataset, GSE25066, which was selected as a neoadjuvant therapy cohort, was also downloaded from the GEO database.

### Identification of Differentially Expressed Platelet-Related Genes

Raw transcriptome count data of 115 TNBC patients and 113 compared normal samples in the TCGA–BRCA cohort were prepared to identify the differentially expressed genes (DEGs). The “edgeR” package was used subsequently to screen out DEGs with a *p* value < 0.05 and the absolute value of the log2 fold change (log2FC) > 1 (Robinson et al., 2010). Based on these DEGs, Kyoto Encyclopedia of Genes and Genomes (KEGG) and gene ontology (GO) analyses were performed (R package “clusterProfiler”) (Yu et al., 2012).

### Identification of Variant Characteristics of Platelet-Related Genes

We applied the “maftools” package to analyze masked somatic mutation information of TNBC patients (Mayakonda et al., 2018). CNV values of the platelet-related genes were screened out and those less than −0.2 were deemed as “loss” while greater than 0.2 were regarded as “gain”. The varied characteristics of platelet-related genes were shown in a circus plot with the help of the “RCircos” package (Zhang et al., 2013).

### Unsupervised Clustering of Platelet-Related DEGs

According to the DEGs, we used the “ConsensusClusterPlus” package to complete consensus clustering (CC) in order to identify the unidentified subtypes of TNBC (Wilkerson and Hayes, 2010). The CC parameter “maxK” was selected as “10,” “clusterAlg” was selected as “pam,” and “distance” was selected as “pearson.” The “gsva” package was applied to perform the single-sample gene set enrichment analysis (ssGSEA), and the “limma” package was used to find out different active pathways among three clusters. The reference database was “h.hallmark.v7.4.symbols.gmt” (Hänzelmann et al., 2013; Ritchie et al., 2015).

### Construction of the Platelet-Related Gene Signature

Univariate Cox regression was utilized to assess whether these genes made a difference to the survival status in both the TCGA cohort and the GSE58812 cohort. To avoid omissions, we adjusted the cutoff *p* value to 0.1. The least absolute shrinkage and selection operator (LASSO) Cox regression method (R package “glmnet”) was further used to shrink the candidates in order to construct the most suitable signature, with the selection of “lambda. min” (Friedman et al., 2010). Ultimately, the model exported the risk score of each patient by the formula below:

$$Risk\;score=\overset{7}{\underset{i=1}{\sum }}\beta i*Ei$$

(βi represents the coefficient index, and Ei represents the gene expression level).

To make plots more intuitionistic, we adjusted the risk score by using a linear transformation. The calculated risk score subtracted the minimum and divided it by the maximum, which mapped these exponentials to the range of 0–1. The formula was as follows:

$$\mathrm{Adjust}\;\mathrm{Risk}\;\mathrm{score=}\frac{Risk\;score-\min\left(Risk\;score\right)}{\max\left(Risk\;score\right)-\min\left(Risk\;score\right)}$$

On the grounds of the median value of the risk score, TNBC patients in each cohort were classified into low-risk and high-risk groups. We used the “stats” R package to perform principal component analysis (PCA). The prognostic difference between the two groups was investigated *via* Kaplan–Meier analysis (R package “survival”). Besides, the “survminer,” “rms,” and “timeROC” R packages were applied to finish receiver operating characteristic (ROC) analysis (Blanche et al., 2013).

### Independent Prognostic Analysis of the Risk Score

We collected the clinical features (age, pathologic T, pathologic N, and stage) of TNBC patients in the TCGA cohort, and they were analyzed together with the risk score by means of univariate and multivariable Cox regression.

### Functional Enrichment Analyses Between Risk Groups

Gene set enrichment analysis (GSEA, https://software.broadinstitute.org/gsea/index.jsp/) was used to find out the different biological functions and signaling pathways between the two groups. We set “c2.cp.kegg.v7.4.symbols.gmt” and “c5.go.bp.v7.4. symbols.gmt” as the reference database, and the cutoff criteria were set to |NES| > 1.5 and Q < 0.25. Gene set variation analysis (GSVA) was employed with the “c2.cp.reactome.v7.4.symbols.gmt” database, and their correlations with the risk score were also calculated by the means of “gsva” and “corrplot” packages (Hänzelmann et al., 2013).

### The Nomogram Establishing

Age, pathologic T, pathologic N, stage, and the risk score were set together. Multivariable Cox regression and stepwise regression analyses were employed to establish a prognostic nomogram. The nomogram plot was shown by the “regplot” package. Calibration plots and decision curve analysis (DCA) were used to evaluate the efficacy of the nomogram (R package “caret” and “rmda”).

### Tumor Immune Microenvironment Analysis

We collected gene expression data in the three cohorts and downloaded the LM22 signatures (Newman et al., 2015). With the help of the online platform CIBERSORTx (https://cibersortx.stanford.edu/), we analyzed whether there was a relationship between the risk groups and the tumor immune microenvironment.

### Statistical Analysis

All statistical analyses were presented *via* R 4.1.0 (https://www.r-project.org/). The Wilcoxon test was employed to evaluate the difference of expression levels of the TNBC samples and normal tissues, as well as low- and high-risk groups. The relationship between the risk groups of patients and response to neoadjuvant therapy was assessed by the chi-square test.

## Results

### Identification of DEGs Between TNBC and Normal Tissue

**FIGURE 2:**
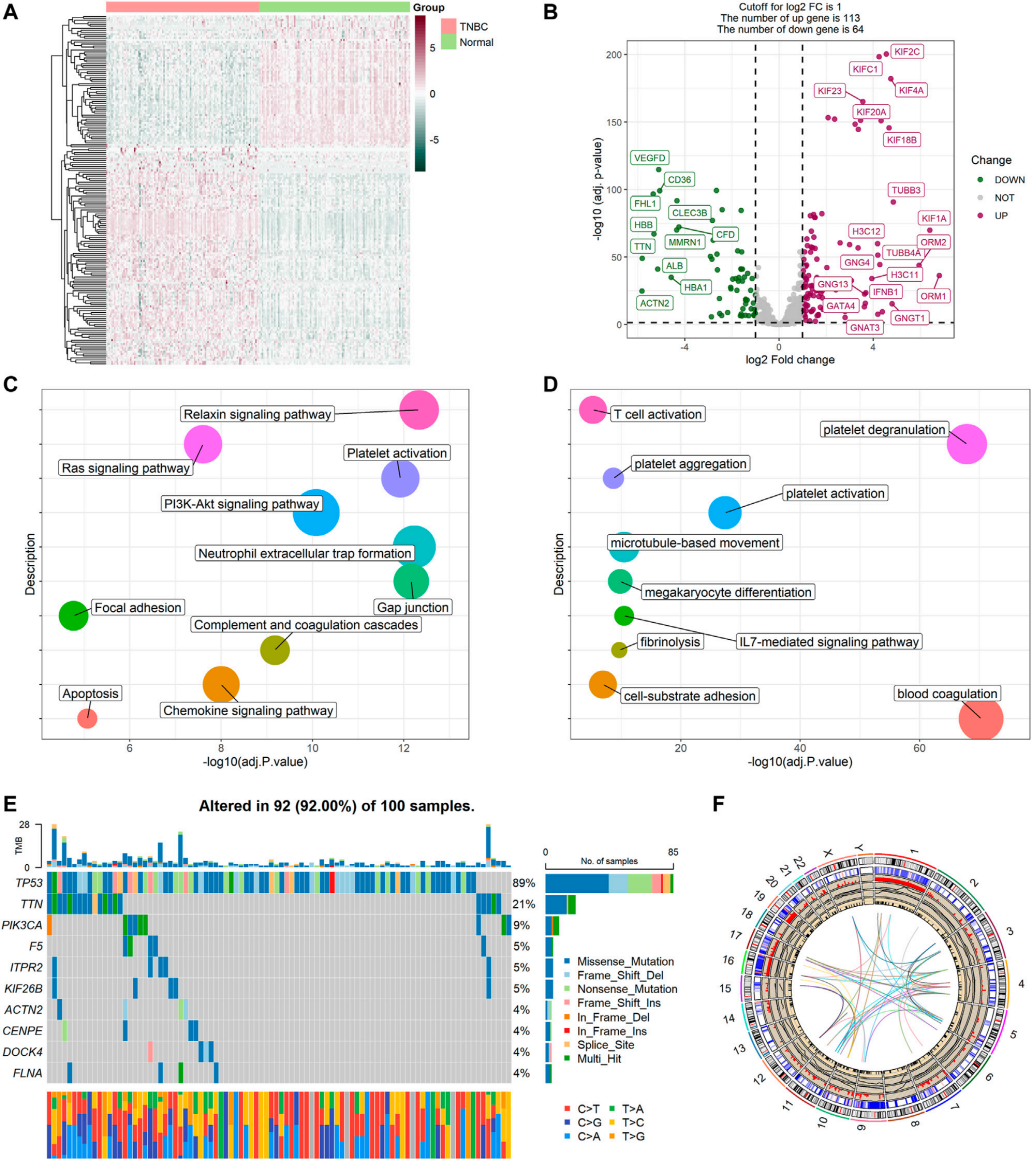
Expressions of the 177 platelet-related DEGs and the functional enrichment analyses. **(A)** Heatmap (atrovirens: low expression level; brick red: high expression level) of the platelet-related DEGs between TNBC (brilliant red) and the normal tissues (brilliant green). **(B)** Volcano plot of the platelet-related DEGs (green: downregulated DEGs; red: upregulated DEGs; gray: unchanged genes). Points with labels are obvious DEGs with *p* value < 0.001 and |log2FC| > 3.5. **(C)** Bubble plot for KEGG enrichment (different colors represent different descriptions, and sizes reflect the enrichment numbers of genes). **(D)** Bubble plot for GO enrichment (biological processes). **(E)** The variation of platelet-related genes in the training cohort. **(F)** The location, expression, CNV values, and correlation of platelet-related genes in TCGA cohort.

The expression levels of 480 platelet-related genes were compared between samples from 115 patients with TNBC and 113 normal samples in the TCGA-BRCA cohort, and 177 DEGs were identified. In the TNBC group, 64 genes were downregulated, whereas 113 other genes were upregulated (Supplementary Table S2). The heatmap of DEGs is shown in Figure 2A, and the volcano plot of DEGs is presented in Figure 2B. We then performed functional enrichment analyses including KEGG and GO, and the results showed that DEGs were directly related to the platelets (Figures 2C,D), including platelet activation, degranulation, and aggregation (Supplementary Table S3).

### Variant Landscape of Platelet-Related Genes

Variations in platelet-related genes were also evaluated in patients with TNBC from the TCGA cohort. The result showed there was approximately 92% (92/100) of TNBC patients who experienced mutations. The top 10 mutations of platelet-related genes are displayed in Figure 2E with TP53 as the most frequent (89%) and nine other mutations ranging in frequency from 4 to 21%. Meanwhile, CNV status analysis indicated frequent alterations in platelet-related genes. We noted that YWHAZ and ZFPM2 possessed the most significant copy number amplification, whereas F2RL2 had the most extensive CNV deletion (Supplementary Figure S1). The location, expression, CNV values, and correlation of each platelet-related gene are shown in Figure 2F.

### TNBC Platelet-Related Subtypes Based on the DEGs

**FIGURE 3:**
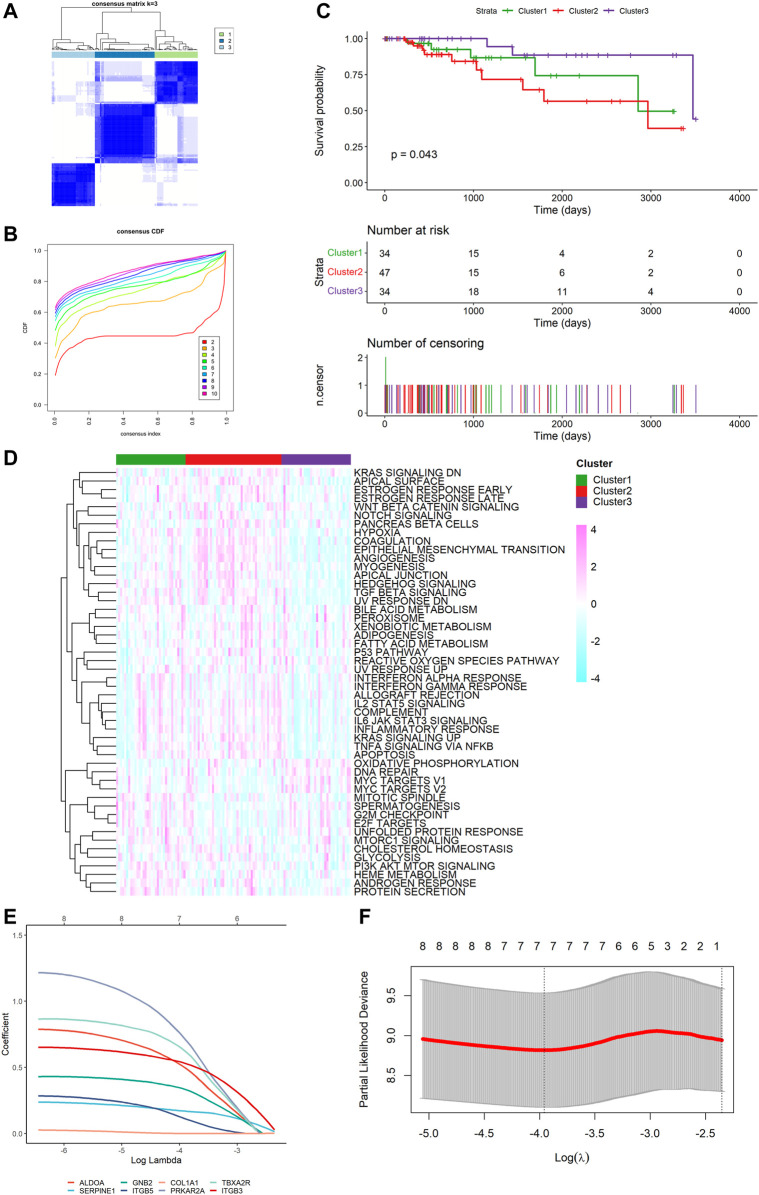
Tumor classification based on the platelet-related DEGs and construction of risk signature in TCGA cohort. **(A)** 115 TNBC patients were divided into three subgroups (k = 3). **(B)** Consensus Cumulative Distribution Function (CDF) Plot under k = 2–10. **(C)** Kaplan–Meier OS curves for the three clusters. **(D)** Heatmap (light blue: low expression level; pink: high expression level) of the hallmark gene set among three clusters (green: cluster 1, red: cluster 2, purple: cluster 3). **(E)** LASSO Cox regression of the 7 model genes. **(F)** Cross-validation for the LASSO Cox regression.

To explore unidentified subtypes of TNBC, we used the expression of platelet-related DEGs to perform CC analysis of the TCGA cohort. We found that when k = 3, the differences among subgroups were the most obvious, which indicated that 115 patients with TNBC could be classified into three clusters (Figures 3A,B). It is worth noting that there were obvious differences between the overall survival (OS) time and the three clusters (*p* = 0.043, Figure 3C). Cluster three was associated with a favorable prognosis, while cluster two was associated with a poor prognosis, and cluster one was between them. However, we found no substantial differences in the clinical features among the three clusters. We then performed ssGSEA and found that different clusters were enriched in certain pathways. For example, the worst-prognosis cluster two was rich in the KRAS signal pathway, angiogenesis, and coagulation, while it was downregulated in DNA repair and the G2M checkpoint compared to the best-prognosis cluster three (Figure 3D).

### Construction of a Prognostic Gene Signature

Survival information was collected and matched with TNBC patients. Univariate Cox regression analysis was performed separately for general filtration. In TCGA, 46 genes met the cutoff of *p* < 0.1 as did 90 genes in GSE58812. The intersection of the two outputs had nine genes (*ALDOA*, *SERPINE1*, *COL1A2*, *GNB2*, *ITGB5*, *COL1A1*, *PRKAR2A*, *TBXA2R*, and *ITGB3*). All were risk factors with hazard ratios (HRs) > 1. By performing the LASSO Cox regression analysis, we constructed a seven-gene signature with a minimum value of lambda (λ) (Figures 3E,F). We investigated the correlation of each model gene (Supplementary Figure S2), using Kaplan–Meier (K-M) analysis to determine their respective influences on the OS time (Supplementary Figure S3) and the Wilcoxon test to examine the model genes’ expression levels between the TNBC tissues and normal samples (Supplementary Figure S4). The results showed that five genes (*ALDOA*, *SERPINE1*, *GNB2*, *ITGB5*, and *ITGB3*) had a significant influence on the OS time (*p* < 0.05), and the expression of each gene was significantly different (*p* < 0.05). The risk score was calculated as follows: risk score = (0.5144062 * ALDOA exp.) + (0.1688905 * SERPINE1 exp.) + (0.3378084 * GNB2 exp.) + (0.1297068 * ITGB5 exp.) + (0.7508113 * PRKAR2A exp.) + (0.6483026 * TBXA2R exp.) + (0.5324253 * ITGB3 exp.).

### Internal Training and External Validation of the Risk Signature

**FIGURE 4:**
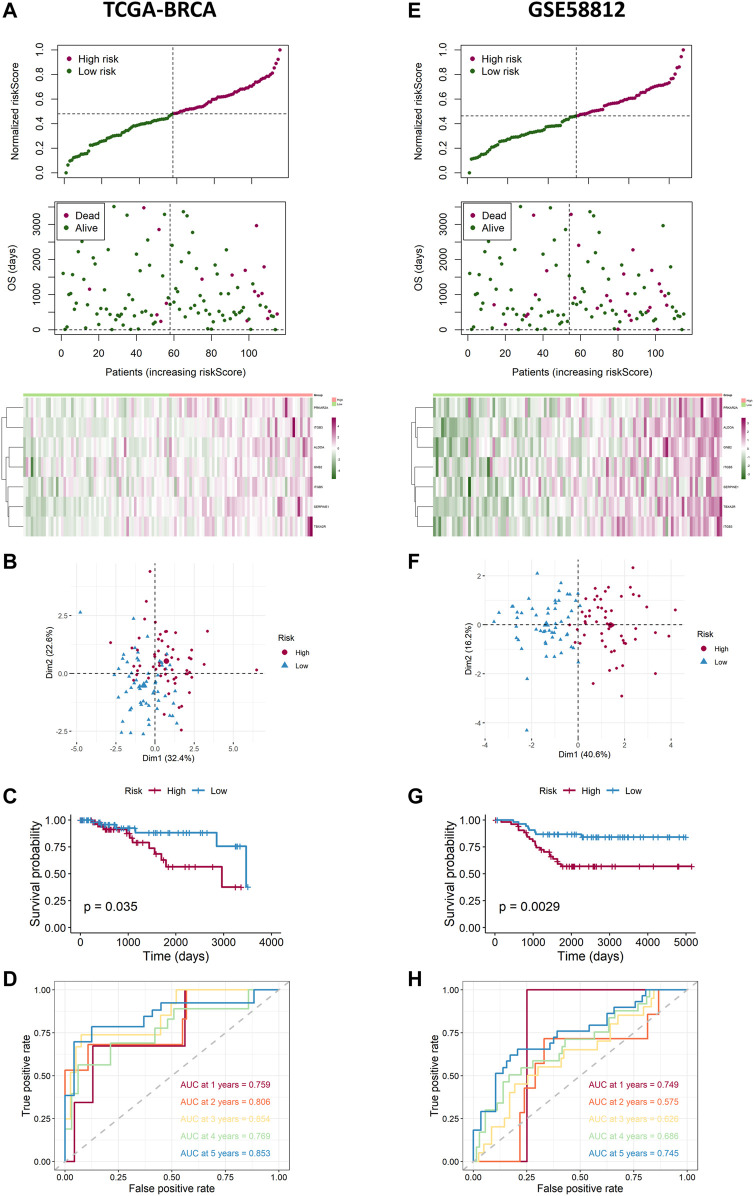
Landscape of adjusted risk score, heatmaps, PCA, Kaplan–Meier analysis, and ROC analysis of the risk signature in the internal set and the external GEO (GSE58812) set. **(A)** Distribution of the adjusted risk score and heatmap in the training TCGA set. **(B)** PCA plot based on the risk groups in the training TCGA set. **(C)** Kaplan–Meier survival analysis of the patients in the training TCGA set. **(D)** ROC curve analysis according to the 1-, 2-, 3-, 4-, and 5-year survival of the AUC value in the training TCGA set. **(E)** Distribution of the adjusted risk score and heatmap in the validation GEO (GSE58812) set. **(F)** PCA plot based on the risk score in the validation GEO (GSE58812) set. **(G)** Kaplan–Meier survival analysis of the patients in the validation GEO (GSE58812) set. **(H)** ROC curve analysis according to the 1-, 2-, 3-, 4-, and 5-year survival of the AUC value in the validation GEO (GSE58812) set.

Based on the median value calculated by the risk score formula above, we divided the 115 patients with TNBC into low- and high-risk groups equally within the TCGA cohort, which we had selected as a training dataset. We found that patients in the high-risk group were more likely to survive as the risk score increased (Figure 4A). Principal component analysis (PCA) showed that this classification performed well (Figure 4B). K-M analysis showed that patients in the low-risk group were more likely to have lower death rates (*p* < 0.05, Figure 4C). In addition, we applied time-dependent ROC analysis to evaluate the efficacy of the prognostic model. The area under the ROC curve (AUC) was 0.759 for 1 year, 0.806 for 2-year, 0.854 for 3-year, 0.769 for 4-year, and 0.853 for 5-year survival (Figure 4D). Consequently, we used GSE58812 as the validation cohort. A higher risk score resulted in shorter survival time (Figure 4E). The two groups were separated using PCA (Figure 4F). K-M analysis also indicated that patients in the low-risk group had longer survival times (*p* < 0.05, Figure 4G). Moreover, ROC curve analysis of GSE58812 indicated that the established prognostic model had excellent predictive efficacy (AUC = 0.749 for 1-year, 0.575 for 2-year, 0.626 for 3-year, 0.686 for 4-year, and 0.745 for 5-year survival) (Figure 4H).

### Independent Prognostic Value of the Risk Signature

**FIGURE 5:**
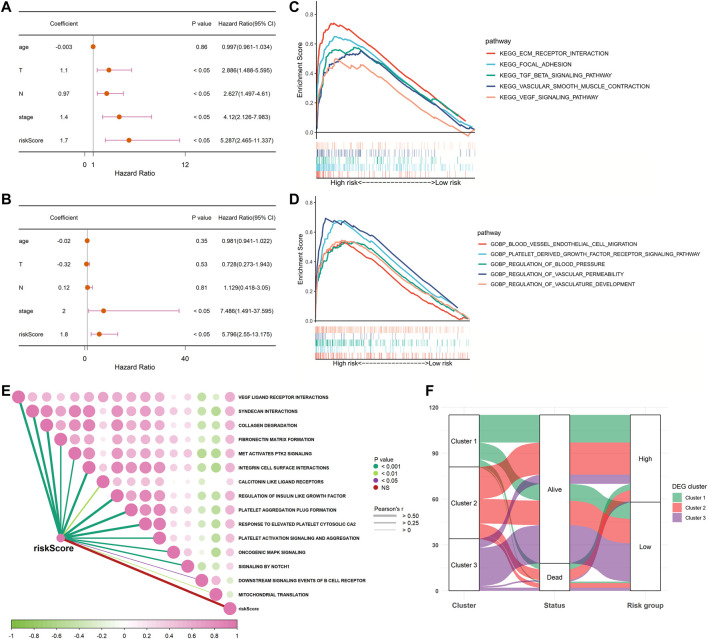
Univariate and multivariate Cox regression analyses for the risk score and GSEA between low- and high-risk groups in TCGA cohort. **(A)** Univariate analysis for TCGA cohort. **(B)** Multivariate analysis for TCGA cohort. **(C,D)** Involved signaling pathways in two risk groups in TCGA cohort. **(E)** Correlation analysis of the risk score and pathways from the reactome gene set. **(F)** Alluvial diagram shows the changes of clusters, vital status, and risk groups in TCGA cohort.

We performed univariate and multivariable Cox regression analyses to explore whether the risk score was an independent prognostic factor. The univariate Cox regression analysis showed that, compared with other features, the risk score was regarded as a risk factor (HR = 5.287, 95% CI: 2.465–11.337, and *p* < 0.05, Figure 5A), predicting poor survival in TCGA cohort. The multivariate analysis further confirmed, after removing confounding factors, the risk score was still an independent risk factor (HR = 5.796, 95% CI: 2.550–13.175, and *p* < 0.05, Figure 5B) for TNBC patients in TCGA cohort.

### Functional Enrichment Analyses Based on the Risk Signature

To further detect the distinction in the signaling pathways and biological processes between the subgroups classified by the risk signature, we applied GSEA to analyze with the criteria of *p* < 0.05 and Q < 0.25. The results are presented in Supplementary Table S4. We observed that the high-risk group was active in the excitation of the extracellular matrix (ECM) receptor interaction, the PDGF receptor signaling pathway, the VEGF signaling pathway, and the transforming growth factor-β (TGF-β) signaling pathway. As for biological processes, the low-risk group was inactive in blood vessel endothelial cell migration, focal adhesion, vascular smooth muscle contraction, regulation of blood pressure, vascular permeability, and vasculature development (Figures 5C,D). Moreover, we also performed an analysis to determine whether there was a relationship between the risk score and pathways derived from the reactome database. This showed that most of the patients in the high-risk group were strongly correlated with platelet-related biological processes, such as platelet activation and platelet aggregation, while negatively correlated with downstream B-cell receptor events and mitochondrial translation (Figure 5E). As shown in Figure 5F, most patients in cluster three were classified into the low-risk group, which was related to better survival outcomes. Collectively, these data suggested that the risk score had a strong correlation with the function and processes of platelets and could effectively predict prognosis.

### Establishment and Assessment of the Nomogram Model

**FIGURE 6:**
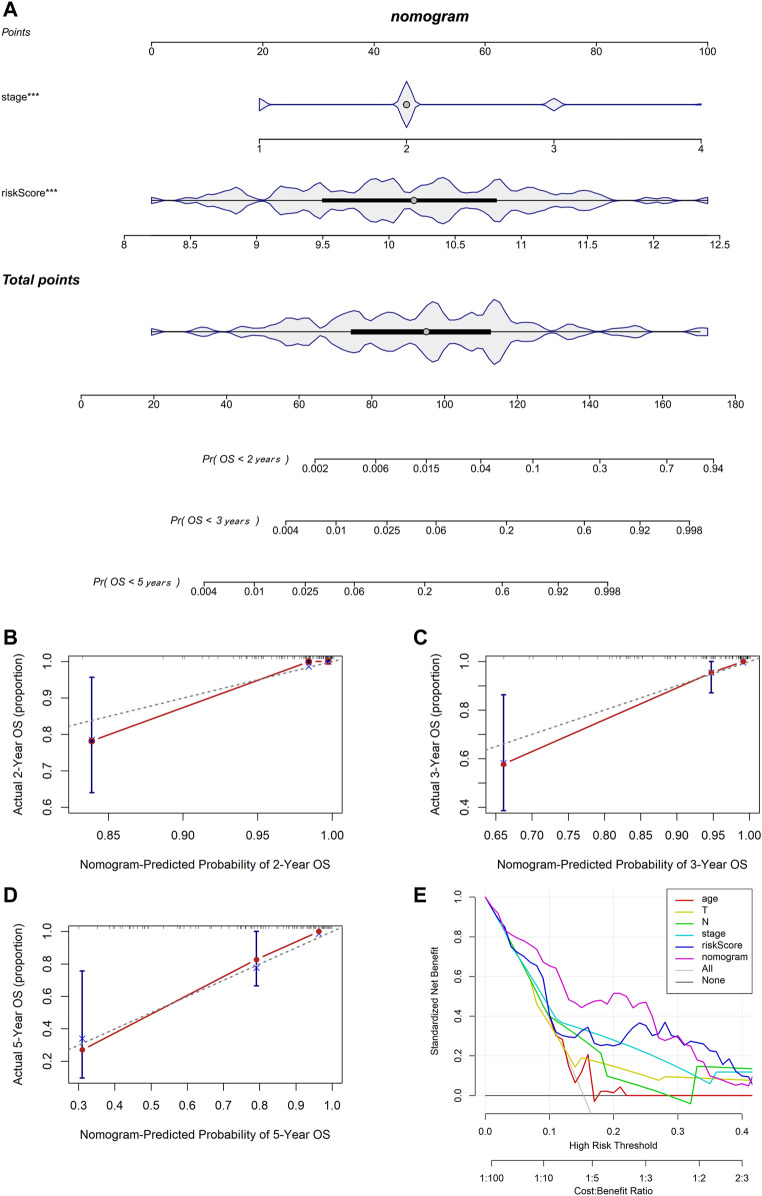
Establishment and assessment of the nomogram based on the 7-gene signature. **(A)** Nomogram for predicting the proportion of patients with 2-, 3-, or 5-year OS (*** means *p* < 0.001). **(B–D)** Calibration plots of the nomogram-predicted probability of 2-, 3-, and 5-year survival in TCGA cohort. **(E)** DCA of the nomogram predicting 2-, 3-, and 5-year OS comparing age, pathologic T, pathologic N, stage, and risk score.

We used multivariable Cox and stepwise regression analyses to establish a nomogram model in TCGA cohort to estimate the probability of two-, three-, and 5-year OS. The stage and risk scores were selected for the model (Figure 6A). The C-index value of the model was 0.916 (95% CI: 0.858–0.973). Calibration curves were used to assess the consistency between the predicted and actual survival rates. The accuracy of this model in predicting the two-, three-, and 5-year survival rates was favorable (Figures 6B–D). Moreover, we performed DCA to confirm a range of threshold probabilities for the model and found that the nomogram model was apparently better than any other predictor applied in this study (Figure 6E).

### Comparison of the Tumor Immune Microenvironment Between Risk Groups

**FIGURE 7:**
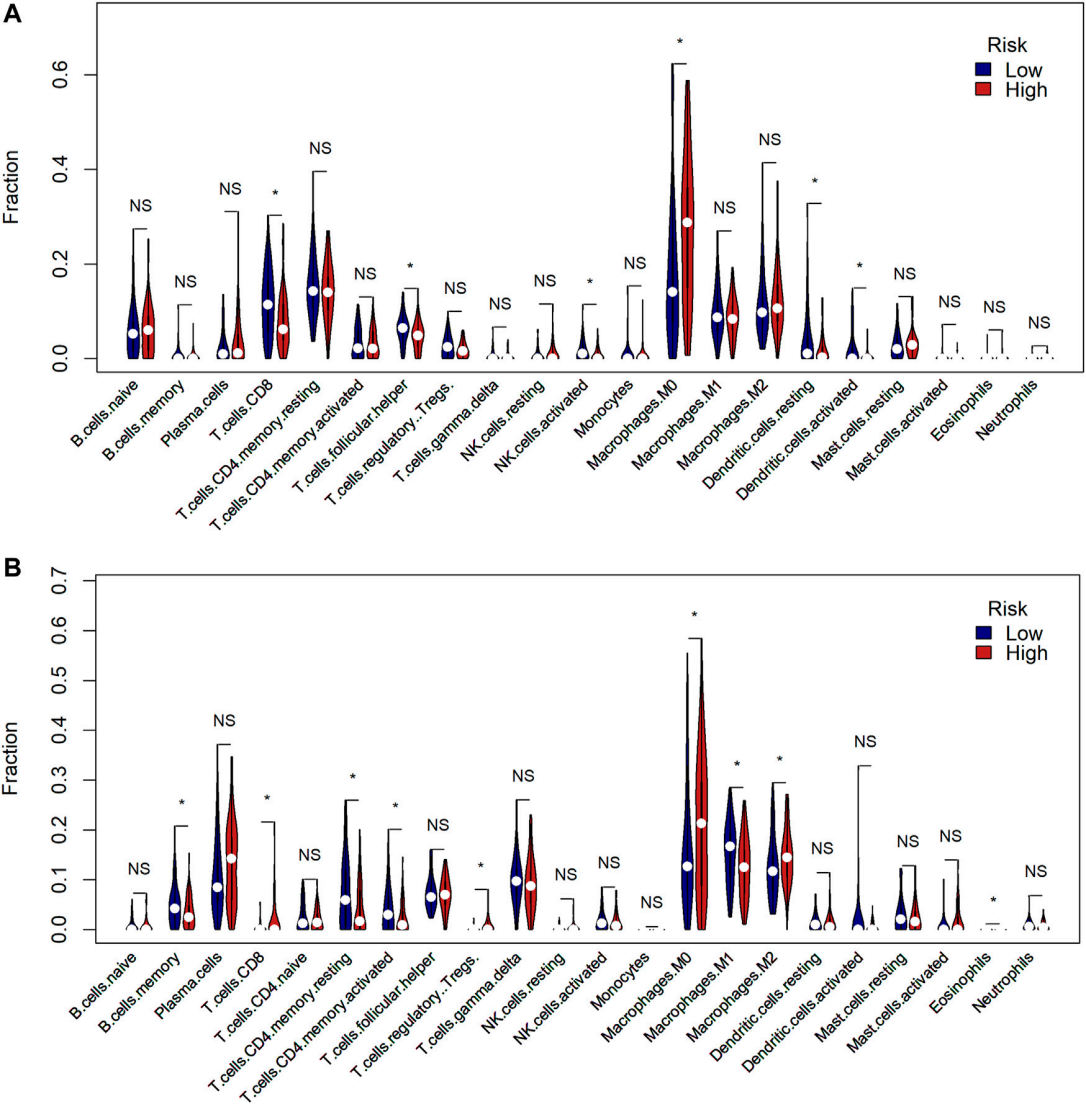
Comparison of the tumor immune microenvironment between risk groups. **(A)** Comparison of the enrichment scores of 22 types of immune cells between low- (blue) and high-risk (red) groups in TCGA cohort (* means *p* < 0.05, NS means no significance). **(B)** Comparison of the enrichment scores of 22 types of immune cells between low- (blue) and high-risk (red) groups in the GEO cohort.

We then proceeded with analysis to identify whether there was a difference in the tumor immune microenvironment between the two risk groups in TCGA and GSE58812 cohorts. We used CIBERSORTx to calculate the enrichment scores of 22 immune-related cells in each sample. In TCGA cohort, the high-risk group frequently had lower levels of immune cell infiltration, especially CD8^+^ T cells, follicular helper T cells, active NK cells, and dendritic cells. However, the expression of macrophage 0 (M0) infiltration was significantly upregulated in the high-risk group (Figure 7A). A similar immune status was found in the GSE58812 cohort; the high-risk group generally had lower levels of infiltration of immune cells, especially memory B cells, memory CD4^+^ T cells, macrophage 1 (M1) cells. However, the levels of infiltration of CD8^+^ T cells, regulatory T cells (Tregs), M0 cells, and macrophage 2 (M2) cells were higher in the high-risk group (Figure 7B).

### Extra Test of the Risk Signature

**FIGURE 8:**
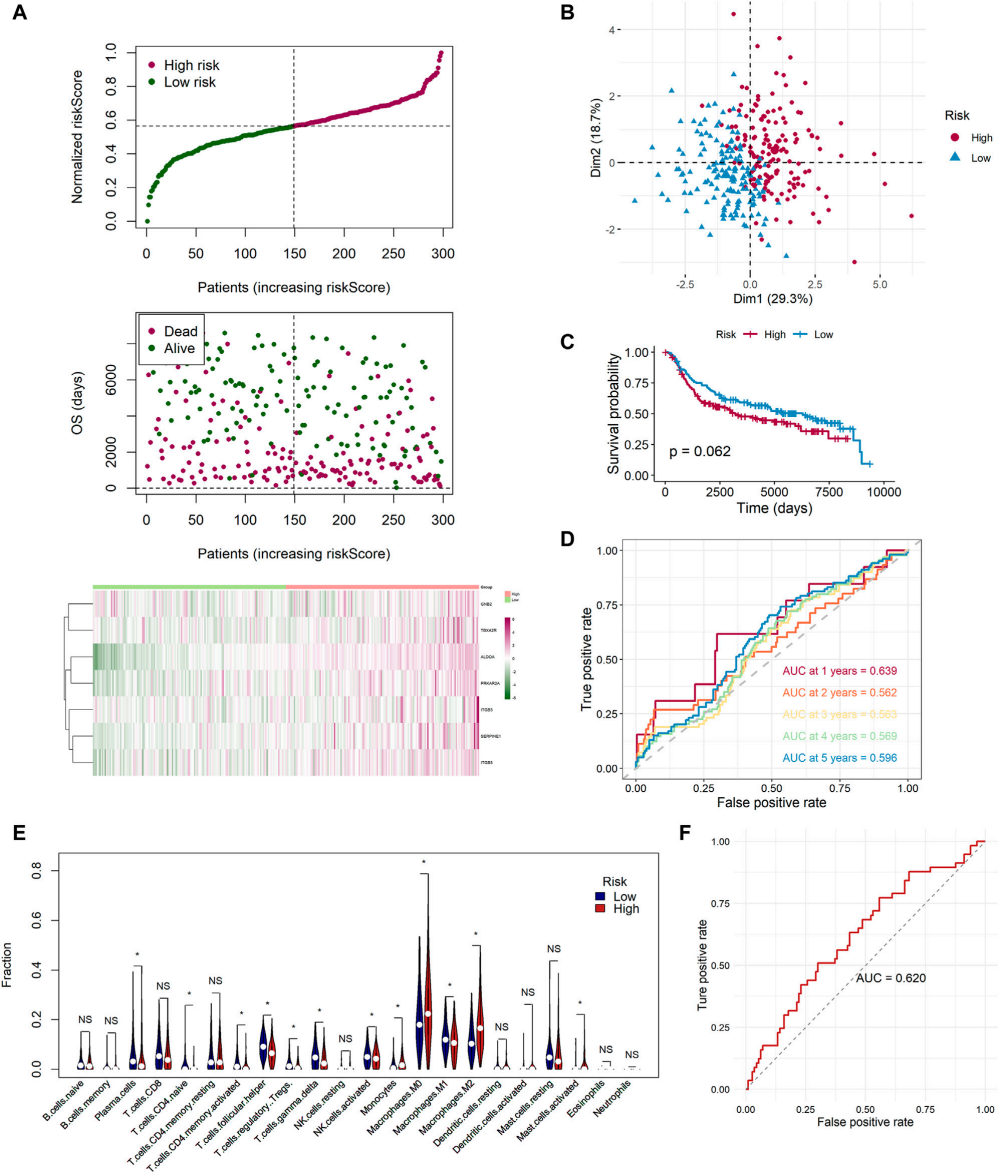
Extra test of the risk signature in the METABRIC cohort and a GEO neoadjuvant therapy cohort (GSE25066). **(A)** Distribution of the adjusted risk score and heatmap in the METABRIC cohort. **(B)** PCA plot based on the risk score in the METABRIC cohort. **(C)** Kaplan–Meier survival analysis of the low- and high-risk group patients in the METABRIC cohort. **(D)** ROC curve analysis according to the 1-, 2-, 3-, 4-, and 5-year survival of the AUC value in the METABRIC cohort. **(E)** Comparison of the enrichment scores of 22 types of immune cells between low- (blue) and high-risk (red) groups in the METABRIC cohort. **(F)** ROC curve analysis for predicting the response of neoadjuvant therapy in the GEO neoadjuvant therapy cohort (GSE25066).

To ensure that the established risk signature was widely applicable, we collected 298 patients with TNBC from the METABRIC dataset, which was used as a test cohort. After calculating the risk scores, the patients were divided into two risk groups. As the risk score increased, patients were more likely to die (Figure 8A). PCA indicated that the classification was distinct (Figure 8B). The K-M analysis showed that patients in the low-risk group lived longer, with a nearly significant *p* value (*p* = 0.062, Figure 8C). ROC curve analysis of the METABRIC cohort revealed that the risk signature was suitable for predicting the prognosis (AUC = 0.639 for 1-year, 0.562 for 2-year, 0.563 for 3-year, 0.569 for 4-year, and 0.596 for 5-year survival) (Figure 8D). With respect to the tumor immune microenvironment, the result was the most similar to the GSE58812 cohort, in which the high-risk group had a lower level of infiltration of active memory T cells and M1 cells, while it had higher levels of infiltration of M0 and M2 cells (Figure 8E).

In addition, we applied the risk signature to GSE25066, which was the neoadjuvant therapy cohort. We sought to identify whether the risk signature was valuable for predicting the curative effect of neoadjuvant therapy in patients with TNBC. A total of 170 TNBC patients were classified into two groups, pathologic complete response (pCR) and non-complete response (nCR), in accordance with the response to neoadjuvant therapy. By utilizing the ROC analysis, we found that the risk scores of 113 samples in the nCR group were higher than the 57 sampled in the pCR group, and the AUC was 0.620, which was noteworthy (Figure 8F). In addition, we applied the chi-square test and found that there were 36 patients with pCR in the low-risk group while only 21 in the high-risk group (*p* = 0.015). The proportion of pCR in the low-risk group was much higher, indicating that these patients could be sensitive to neoadjuvant therapy (Supplementary Figure S5).

## Discussion

Regarded as the most invasive breast cancer subtype, TNBC lacks effective therapeutic targets and accurate efficacy prediction models. In this study, we first applied bioinformatics methods to explore the relationship between platelets and TNBC. We constructed a seven-gene risk signature in the TCGA cohort *via* univariate Cox analysis and LASSO Cox regression analysis, and the model was shown to perform well in the external validation dataset GSE58812. Some classical signaling pathways and platelet-related biological processes were found to be active in high-risk groups. We built a nomogram including clinical characteristics and risk scores, and the results showed that it performed well. The differences in the tumor immune microenvironment between the two risk groups were compared, and universally upregulated levels of M0 cells and downregulated levels of M1 and M2 cells were found in the high-risk group compared with the low-risk group. Finally, we added a test set downloaded from the METABRIC to ensure that the established risk signature was widely applicable, and we obtained a good outcome. Moreover, we applied the model to a neoadjuvant therapy cohort and found that the model could be useful for predicting the efficacy of neoadjuvant therapy in patients with TNBC.

Platelets are one of the three main types of blood cells in the human body and act as a double-edged sword. On the one hand, platelets are involved in the angiogenesis and metastasis of tumors (Menter et al., 2017). On the other hand, inhibition of platelet activity and quantity could be a novel therapeutic target (Yeung et al., 2018). Our study built a signature covering seven platelet-related genes (*ALDOA*, *SERPINE1*, *GNB2*, *ITGB5*, *PRKAR2A*, *TBXA2R*, and *ITGB3*) and found that it had the ability to predict OS in TNBC patients. Aldolase A (ALDOA) is an aldolase isozyme (ALDOA, ALDOB, and ALDOC). ALDOA participates in many biological processes and cellular functions, including cell morphology, motor regulation, muscle maintenance, actin filament constitution, and ATP biosynthesis (Carr and Knull, 1993; Kusakabe et al., 1997; Kajita et al., 2001). It is worth noting that ALDOA is highly expressed in various cancers, such as colorectal cancer, hepatocellular carcinomas, and pancreatic cancer (Peng et al., 2012; Ji et al., 2016). In addition, a deficiency of ALDOA is related to hemolytic anemia (Yao et al., 2004). In our study, ALDOA was highly expressed in TNBC samples, and a high expression of ALDOA was not effective for prolonging survival time, which was in agreement with previous studies. SERPINE1, also called plasminogen activator inhibitor 1 (PAI1), mediates the inhibition of fibrin degradation (Placencio and DeClerck, 2015). High concentrations of SERPINE1 are associated with thrombosis (Corduan et al., 2015). SERPINE1 is overexpressed in numerous cancers, especially breast cancer. Previous studies confirmed that upregulation of SERPINE1 predicted worse overall survival, greater possibility of metastasis, and poorer responses to chemotherapy, which was consistent with our study results (Duffy et al., 2014). G Protein Subunit-β2 (GNB2) is a protein-coding gene that is responsible for the formation of beta subunits (Blatt et al., 1988). GNB2 has been reported to be frequently mutated and upregulated in many hematological neoplasms, and a lower expression of GNB2 could reduce the proliferation potential of tumor cells (Kotani et al., 2019). Our study confirmed that GNB2 was a risk gene. Integrin-β5 (ITGB5) belongs to the integrin family, which regulates many biological functions in tumors such as proliferation, adhesion, migration, and invasion (Lin et al., 2018). The role of ITGB5 in angiogenesis has also been demonstrated (Su et al., 2007). Although GNB2 was a risk factor for survival in our study, interestingly, the expression of GNB2 was lower in TNBC samples than in normal tissues. Given the sparse data from TNBC and the contradictory results in other tumors, further studies are needed. PRKAR2A codes for protein kinase A. Previous studies have shown that PRKAR2A deficiency predisposes patients to hematopoietic malignancies (Saloustros et al., 2015). In addition, PRKAR2A has been found to regulate the response of cancer cells to chemotherapy, which might be the reason why our established model helped predict the curative effect of neoadjuvant therapy in TNBC patients (Zynda et al., 2014). The thromboxane A2 receptor (TBXA2R) is a specific coordinator of the T prostanoid receptor (TPR), which is associated with the platelet activity. The variants of the TBXA2R gene cause bleeding and increased metastasis in multiple cancers (Mundell and Mumford, 2018; Pulley et al., 2018). Our study also confirmed that TBXA2R was highly expressed in TNBC samples and was a risk factor for survival. Integrin-β3 (ITGB3), also named CD61 or GP3A, is a member of the integrin family and has been widely studied by scientists. ITGB3 acts as a receptor and participates in forming the tumor stromal and immune microenvironment, as well as maintaining tumor stemness (Zhu et al., 2019). In addition, ITGB3 can mediate extracellular vesicles to facilitate intercellular communication in BC cells (Fuentes et al., 2020). The expression level of ITGB3 was lower in the TNBC samples according to our study, which was similar to that of ITGB5.

Tumor cells can survive because the tumor microenvironment provides a haven for them to escape immune surveillance and drug interference (Zou et al., 2019). High infiltration levels of immune cells can enhance the efficacy of neoadjuvant therapy for BC patients, and the levels of antitumor-infiltrating immune cells in the high-risk group would be low, which indicates holistic damage of immune functions (Zou et al., 2020; Zou et al., 2021). Surprisingly, among the three cohorts, the expressions of M0 and M2 cells were upregulated, while the expression of M1 cells was downregulated in the high-risk group. M0 cells are resting macrophages that can be polarized into two different phenotypes, M1 and M2. Both M1 and M2 cells are closely associated with inflammatory responses; M1 cells are mainly involved in pro-inflammatory responses, while M2 cells mainly participate in anti-inflammatory responses (Mehla and Singh, 2019). Theoretically, the increase in platelets is correlated with anti-inflammation, which was similar to our results. Previous studies have shown that the abundance of M1 cells represents a better outcome, while the enrichment of M2 cells indicates a poorer prognosis in the TNBC microenvironment (Zheng et al., 2020b). We hypothesized that the phenotypic shift of the M2 subtype toward the M1 subtype might be a strategy to overcome the early phases of inflammation and immunotherapy.

The established seven-gene signature and nomogram reflected excellent performance in internal and external cohorts; however, it had the following shortcomings. First, the seven genes mentioned above were all risk factors for survival; the interaction of each other during platelet activation needs further investigation, and the roles of some of the candidates, such as PRKAR2A, that occur in TNBC have not been revealed, which might be an important point for further research. Second, the signature lacked verification of large-scale prospective trials. Third, TCGA cohort was composed of gene sequencing data, while the GSE58812 and METABRIC cohorts were gene chip data, indicating that the results originating from external cohorts might not fully reveal actual prognostic efficacy in TNBC. Ultimately, the detailed mechanisms have not been explored at the cellular and molecular levels.

In summary, we presented a novel platelet-related gene signature as a practical tool for patients with TNBC, which can offer an independent value in the assessment of clinical outcomes.

## Data Availability

The datasets presented in this study can be found in online repositories. The names of the repository/repositories and accession number(s) can be found in the article/Supplementary Material.

## Supplementary Material

Supplementary Figure S1 CNV status analysis of platelet-related genes in TCGA cohort (Top 20 mutation frequency).

Supplementary Figure S2 A correlogram of each model genes. Pearson’s correlation coefficients (r) for all model genes are given in the plot. The areas of the sectors are related to the r. Pink indicates a positive correlation and green indicates a negative correlation.

Supplementary Figure S3 Kaplan-Meier survival analysis of each model genes in TCGA cohort (blue: low-expression group; red: high-expression group).

Supplementary Figure S4 Wilcoxon test of expression levels of each model genes between TNBC tissues (red) and normal samples (green) (* means *P* < 0.05).

Supplementary Figure S5 The correlation of risk groups with the neoadjuvant therapy efficacy in the GEO neoadjuvant therapy cohort (GSE25066, red: nCR; blue: pCR).

Supplementary Table S1 The information of 480 genes related to platelets.

Supplementary Table S2 177 platelet-related DEGs between TNBC and normal tissues.

Supplementary Table S3 Functional enrichment analyses based on the DEGs between TNBC and normal tissues. Table S4. GSEA based on the subgroups categorized by the risk signature.

Supplementary Table S4 GSEA based on the subgroups categorized by the risk signature.
