# Supplementary material for: A Novel Platelet-Related Gene Signature for Predicting the Prognosis of Triple-Negative Breast Cancer
Source: Front Cell Dev Biol. 2022 Jan 12;9:795600. doi: 10.3389/fcell.2021.795600 (PMC8790231; doi:10.3389/fcell.2021.795600)
Supplement: Supplementary file 1 [file DataSheet1.docx]

Supplementary Material

# Supplementary Figures and Tables

## Supplementary Figures


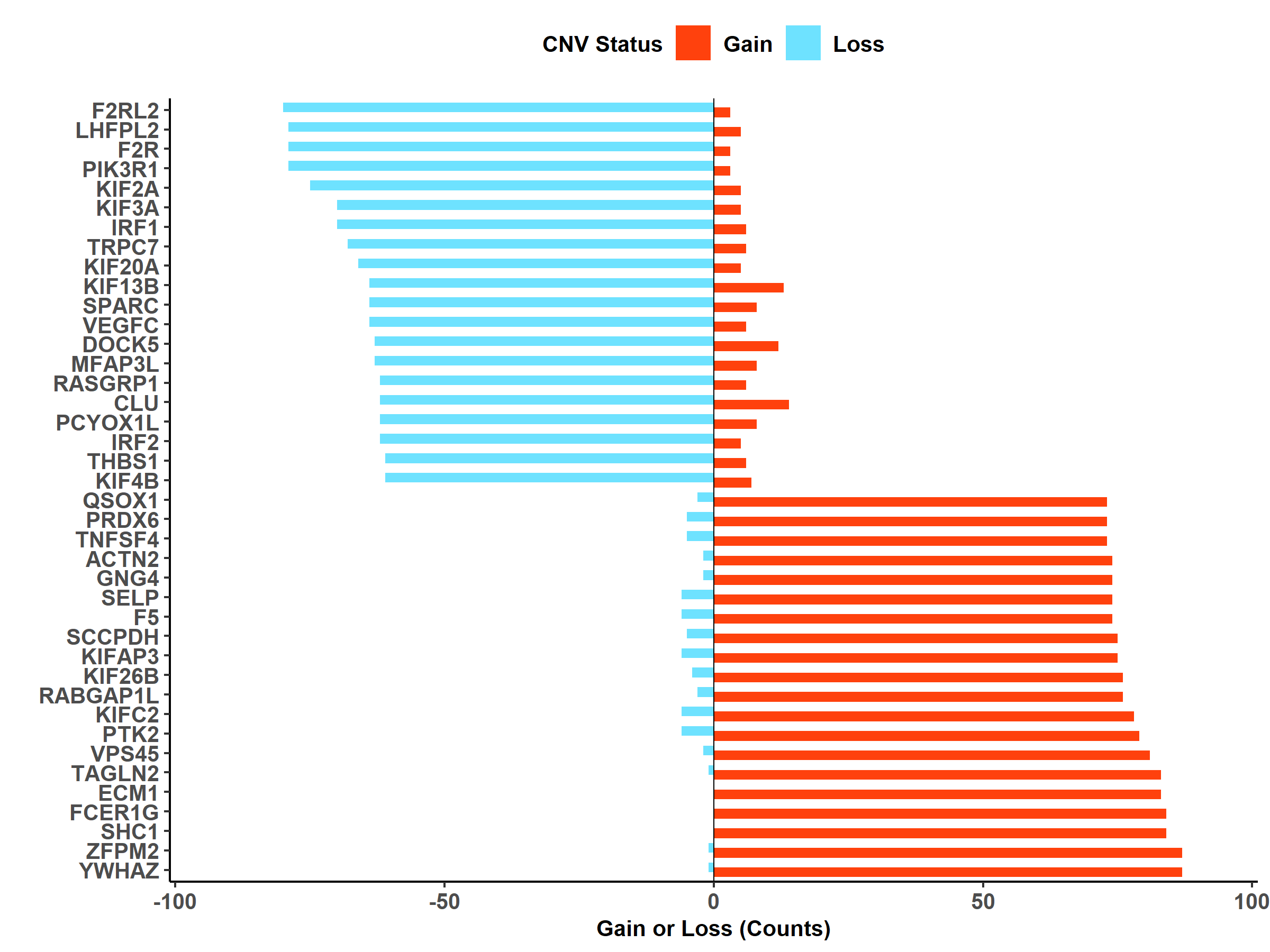


**Supplementary Figure 1.** CNV status analysis of platelet-related genes in TCGA cohort (Top 20 mutation frequency).


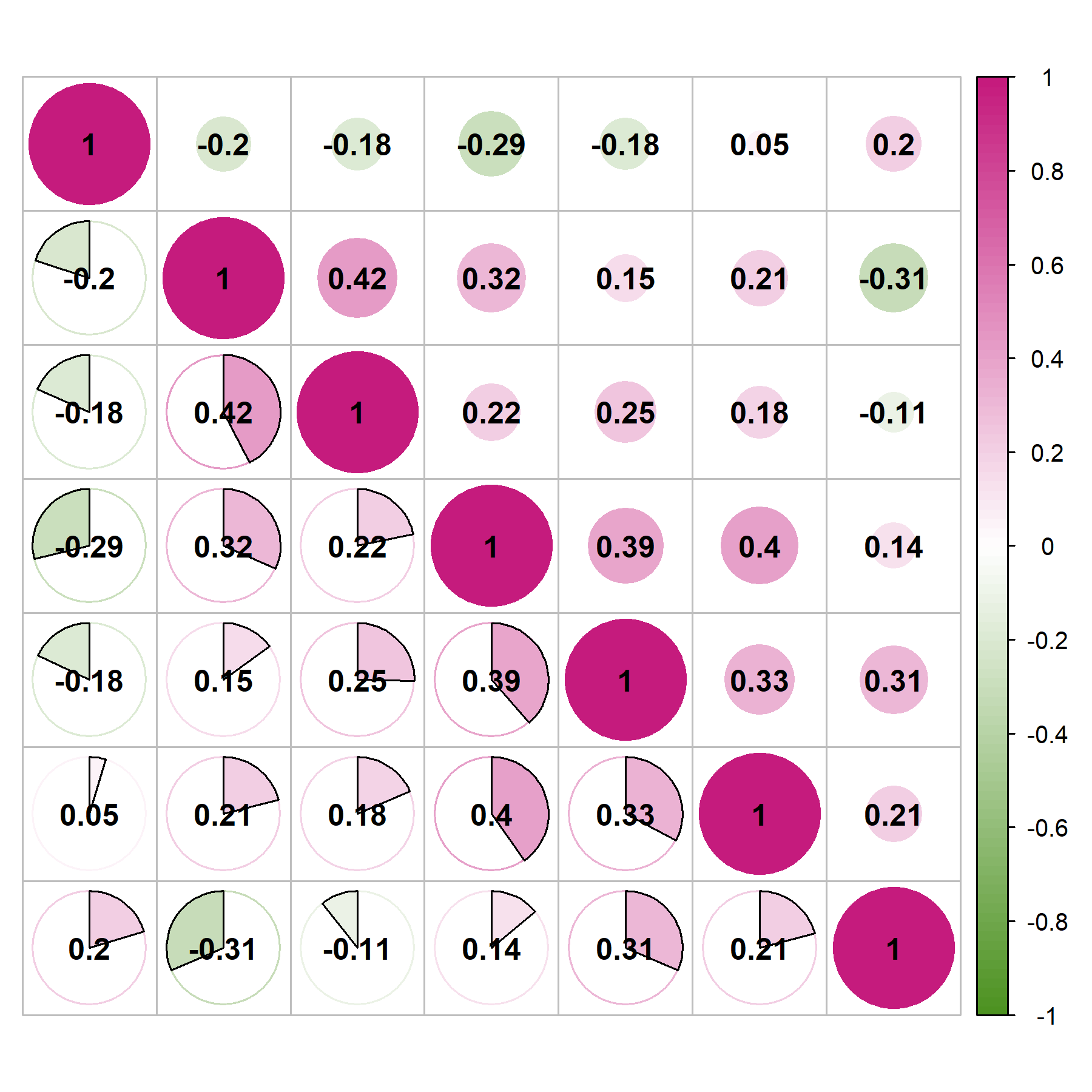


**Supplementary Figure 2.** A correlogram of each model genes. Pearson’s correlation coefficients (r) for all model genes are given in the plot. The areas of the sectors are related to the r. Pink indicates a positive correlation and green indicates a negative correlation.


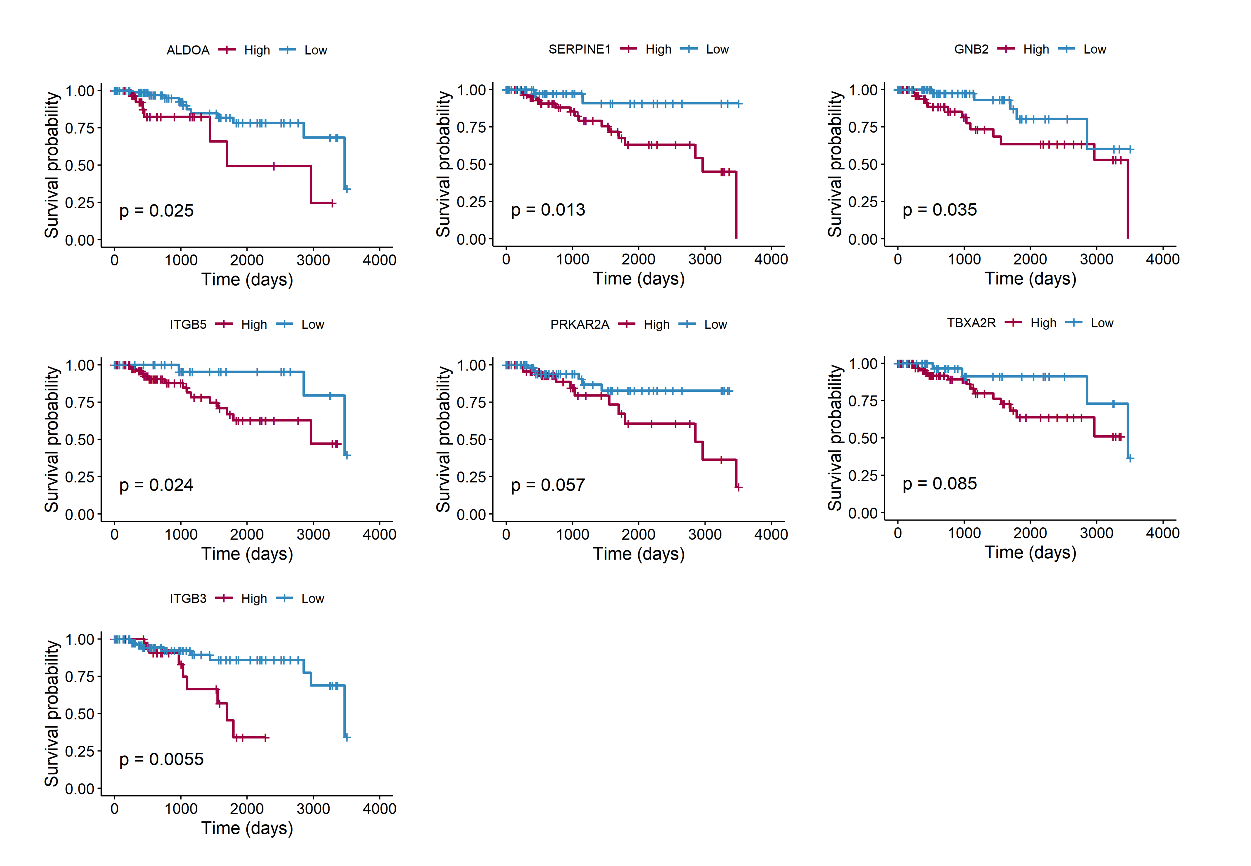


**Supplementary Figure 3.** Kaplan-Meier survival analysis of each model genes in TCGA cohort (blue: low-expression group; red: high-expression group).


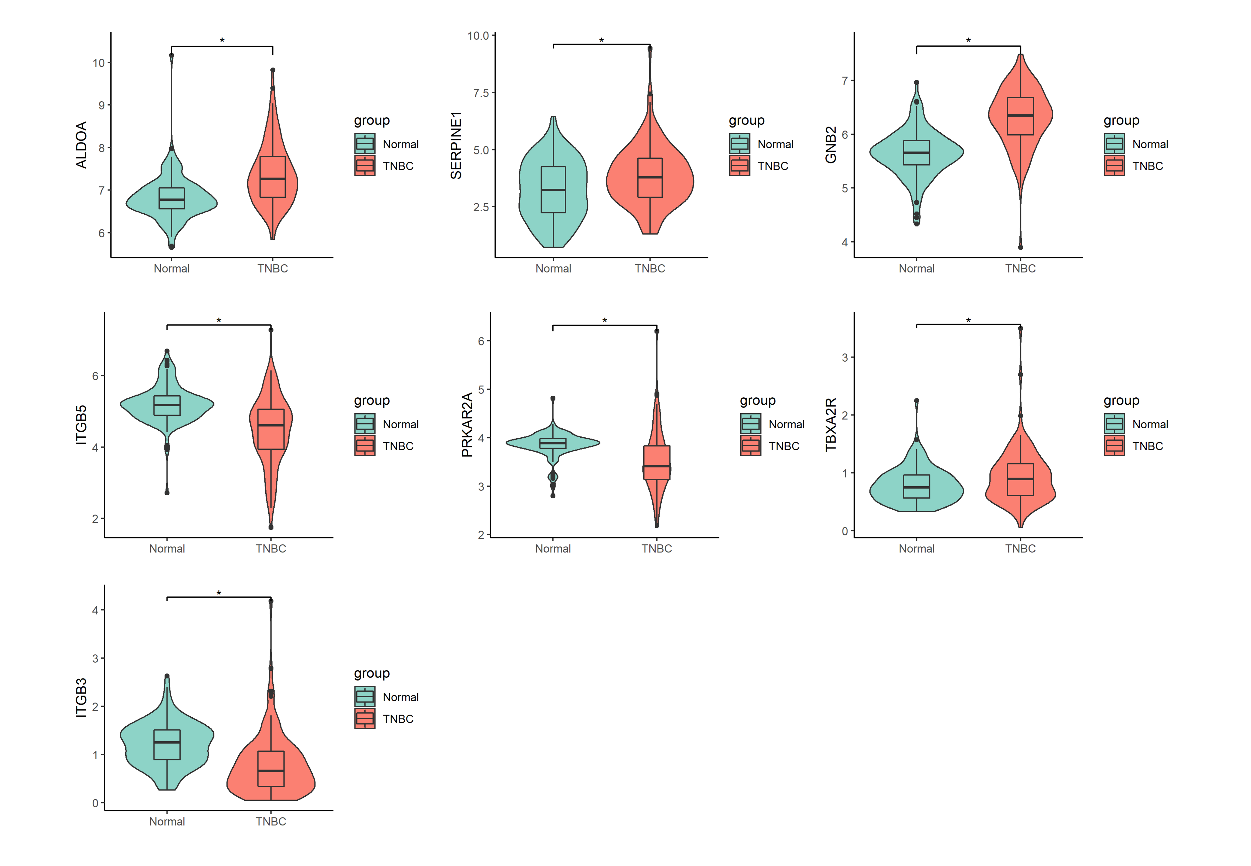


**Supplementary Figure 4.** Wilcoxon test of expression levels of each model genes between TNBC tissues (red) and normal samples (green) (* means P < 0.05).


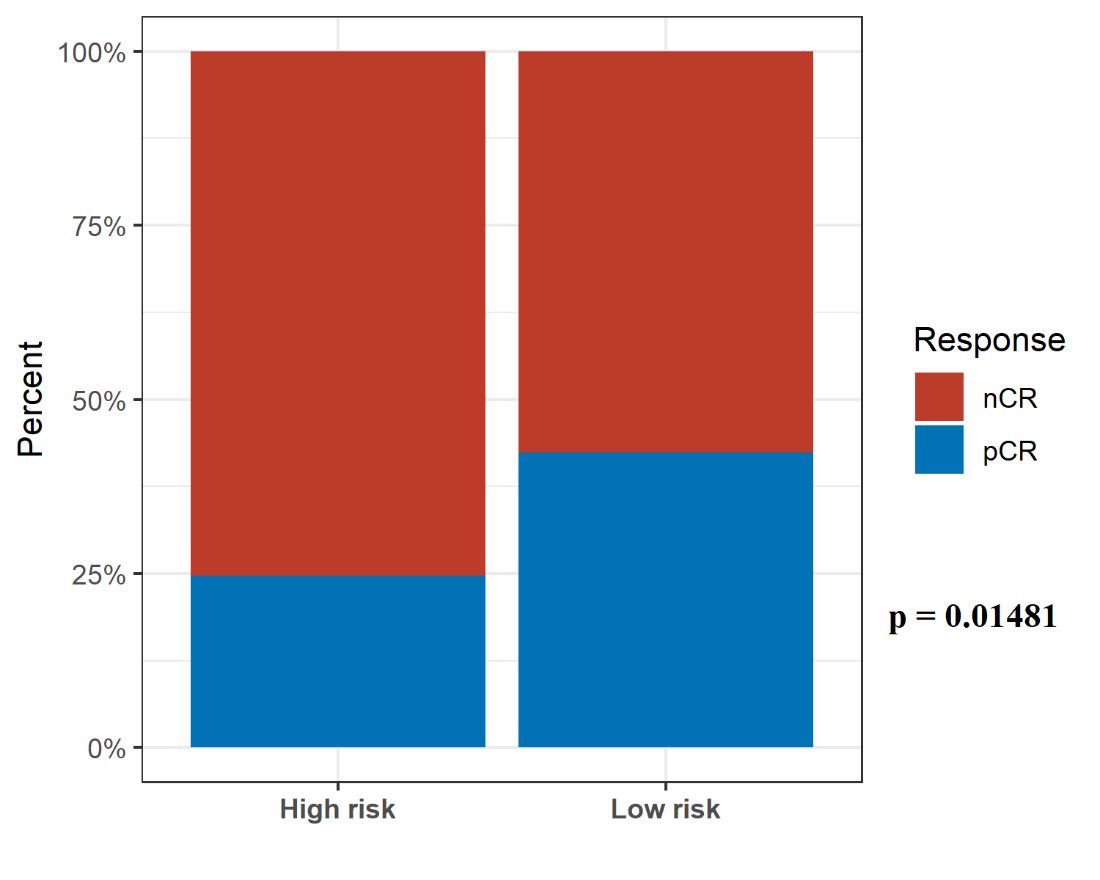


**Supplementary Figure 5.** The correlation of risk groups with the neoadjuvant therapy efficacy in the GEO neoadjuvant therapy cohort (GSE25066, red: nCR; blue: pCR).

## Supplementary Tables

**Supplementary Table 1.** The information of 480 genes related to platelets.

| Gene symbols | | | | | | | |
| --- | --- | --- | --- | --- | --- | --- | --- |
| A1BG | A2M | AAMP | ABCC4 | ABHD12 | ABHD6 | ABL1 | ACTB |
| ACTN1 | ACTN2 | ACTN4 | ADRA2A | ADRA2B | ADRA2C | AHSG | AK3 |
| AKAP1 | AKAP10 | AKT1 | ALB | ALDOA | ANXA5 | APBB1IP | APLP2 |
| APOA1 | APOH | APOOL | APP | ARRB1 | ARRB2 | ASAH1 | BCAR1 |
| BEX3 | BRPF3 | CABLES1 | CABLES2 | CALM1 | CALU | CAP1 | CAPZA1 |
| CAPZA2 | CAPZB | CARMIL1 | CBX5 | CCL5 | CD109 | CD36 | CD63 |
| CD9 | CDC37L1 | CDC42 | CDK2 | CDK5 | CENPE | CFD | CFL1 |
| CHID1 | CLEC1B | CLEC3B | CLU | COL1A1 | COL1A2 | CRK | CSK |
| CTSA | CTSW | CTTN | CXCL5 | CYB5R1 | CYRIB | DAGLA | DAGLB |
| DAPP1 | DGKA | DGKB | DGKD | DGKE | DGKG | DGKH | DGKI |
| DGKK | DGKQ | DGKZ | DOCK1 | DOCK10 | DOCK11 | DOCK2 | DOCK3 |
| DOCK4 | DOCK5 | DOCK6 | DOCK7 | DOCK8 | DOCK9 | ECM1 | EGF |
| EHD1 | EHD2 | EHD3 | EIF2AK1 | ENDOD1 | F13A1 | F2 | F2R |
| F2RL2 | F2RL3 | F5 | F8 | FAM3C | FCER1G | FERMT3 | FGA |
| FGB | FGG | FHL1 | FLNA | FN1 | FYN | GAS6 | GATA1 |
| GATA2 | GATA3 | GATA4 | GATA5 | GATA6 | GLA | GNA11 | GNA12 |
| GNA13 | GNA14 | GNA15 | GNAI1 | GNAI2 | GNAI3 | GNAQ | GNAS |
| GNAT3 | GNB1 | GNB2 | GNB3 | GNB4 | GNB5 | GNG10 | GNG11 |
| GNG12 | GNG13 | GNG2 | GNG3 | GNG4 | GNG5 | GNG7 | GNG8 |
| GNGT1 | GNGT2 | GP1BA | GP1BB | GP5 | GP6 | GP9 | GRB2 |
| GTPBP2 | H2AC6 | H2BC21 | H3-3A | H3-3B | H3C1 | H3C10 | H3C11 |
| H3C12 | H3C13 | H3C14 | H3C15 | H3C2 | H3C3 | H3C4 | H3C6 |
| H3C7 | H3C8 | HABP4 | HBA1 | HBB | HBD | HBE1 | HBG1 |
| HBG2 | HDAC1 | HDAC2 | HGF | HMG20B | HRG | HSPA5 | IFNA1 |
| IFNA10 | IFNA13 | IFNA14 | IFNA16 | IFNA17 | IFNA2 | IFNA21 | IFNA4 |
| IFNA5 | IFNA6 | IFNA7 | IFNA8 | IFNB1 | IGF1 | IGF2 | IRF1 |
| IRF2 | ISLR | ITGA2B | ITGB3 | ITGB5 | ITIH3 | ITIH4 | ITPK1 |
| ITPR1 | ITPR2 | ITPR3 | JAK2 | JMJD1C | KDM1A | KIF11 | KIF12 |
| KIF13B | KIF15 | KIF16B | KIF18A | KIF18B | KIF19 | KIF1A | KIF1B |
| KIF1C | KIF20A | KIF20B | KIF21A | KIF21B | KIF22 | KIF23 | KIF25 |
| KIF26A | KIF26B | KIF27 | KIF2A | KIF2B | KIF2C | KIF3A | KIF3B |
| KIF3C | KIF4A | KIF4B | KIF5A | KIF5B | KIF6 | KIF9 | KIFAP3 |
| KIFC1 | KIFC2 | KLC1 | KLC2 | KLC3 | KLC4 | KNG1 | LAMP2 |
| LAT | LCK | LCP2 | LEFTY2 | LEPROT | LGALS3BP | LHFPL2 | LY6G6F |
| LYN | MAFF | MAFG | MAFK | MAGED2 | MANF | MAP3K7CL | MAPK1 |
| MAPK14 | MAPK3 | MAX | MFAP3L | MFN1 | MFN2 | MGLL | MICAL1 |
| MLH3 | MMD | MMRN1 | MPIG6B | MPL | MPP1 | MYB | MYLK |
| NAP1L1 | NFE2 | NHLRC2 | NRGN | ODC1 | OLA1 | ORM1 | ORM2 |
| P2RY1 | P2RY12 | PCDH7 | PCYOX1L | PDGFA | PDGFB | PDLIM1 | PDPK1 |
| PDPN | PECAM1 | PF4 | PF4V1 | PFN1 | PGRMC1 | PHACTR2 | PHF21A |
| PIK3CA | PIK3CB | PIK3CG | PIK3R1 | PIK3R2 | PIK3R3 | PIK3R5 | PIK3R6 |
| PIP4K2A | PLA2G4A | PLCG2 | PLEK | PLG | PPBP | PPIA | PPM1A |
| PRDX6 | PRKACA | PRKACB | PRKACG | PRKAR1A | PRKAR1B | PRKAR2A | PRKAR2B |
| PRKCA | PRKCB | PRKCD | PRKCE | PRKCG | PRKCH | PRKCQ | PRKCZ |
| PROS1 | PRUNE1 | PSAP | PTGS1 | PTK2 | PTPN1 | PTPN11 | PTPN12 |
| PTPN6 | QSOX1 | RAB27B | RAB31 | RAB5A | RABGAP1L | RAC1 | RAC2 |
| RACGAP1 | RAD51B | RAD51C | RAF1 | RAP1A | RAP1B | RAPGEF3 | RAPGEF4 |
| RARRES2 | RASGRP1 | RASGRP2 | RBSN | RCOR1 | RGS10 | RHOA | RHOB |
| RHOG | RNF11 | RSU1 | RUFY1 | RYBP | SCCPDH | SCG3 | SELENOP |
| SELP | SERPINA1 | SERPINA3 | SERPINA4 | SERPINE1 | SERPINF2 | SERPING1 | SH2B1 |
| SH2B2 | SH2B3 | SHC1 | SIN3A | SNCA | SNN | SOD1 | SOS1 |
| SPARC | SPP2 | SRC | SRGN | STX4 | STXBP2 | STXBP3 | SYK |
| SYTL4 | TAGLN2 | TAX1BP3 | TBXA2R | TEX264 | TF | TGFB1 | TGFB2 |
| TGFB3 | THBS1 | THPO | TIMP1 | TIMP3 | TLN1 | TMEM140 | TMSB4X |
| TMX3 | TNFSF4 | TOR4A | TP53 | TPM1 | TRPC3 | TRPC6 | TRPC7 |
| TSC22D1 | TTN | TUBA1A | TUBA1B | TUBA1C | TUBA3C | TUBA3D | TUBA3E |
| TUBA4A | TUBA4B | TUBA8 | TUBAL3 | TUBB1 | TUBB2A | TUBB2B | TUBB3 |
| TUBB4A | TUBB4B | TUBB6 | TUBB8 | TUBB8B | VAV1 | VAV2 | VAV3 |
| VCL | VEGFA | VEGFB | VEGFC | VEGFD | VPS45 | VTI1B | VWF |
| WDR1 | WEE1 | WIPF1 | YPEL5 | YWHAZ | ZFPM1 | ZFPM2 | ZNF185 |

**Supplementary Table 2.** 177 platelet-related DEGs between TNBC and normal tissues.

| Gene | log2FC | logCPM | LR | P. val | P. adj |
| --- | --- | --- | --- | --- | --- |
| KIF2C | 4.54 | 9.15 | 927.40 | <0.001 | <0.01 |
| KIFC1 | 4.24 | 9.37 | 916.79 | <0.001 | <0.01 |
| KIF4A | 4.74 | 8.79 | 841.78 | <0.001 | <0.01 |
| KIF23 | 3.56 | 8.91 | 762.86 | <0.001 | <0.01 |
| CENPE | 3.46 | 8.36 | 726.57 | <0.001 | <0.01 |
| TUBA1C | 2.08 | 10.57 | 707.40 | <0.001 | <0.01 |
| RACGAP1 | 2.35 | 9.32 | 701.90 | <0.001 | <0.01 |
| KIF15 | 3.46 | 7.72 | 697.86 | <0.001 | <0.01 |
| KIF20A | 4.33 | 8.96 | 696.86 | <0.001 | <0.01 |
| KIF11 | 3.22 | 9.22 | 684.17 | <0.001 | <0.01 |
| KIF18B | 4.67 | 7.82 | 671.82 | <0.001 | <0.01 |
| KIF18A | 3.36 | 7.27 | 666.17 | <0.001 | <0.01 |
| VEGFD | -5.11 | 8.03 | 529.10 | <0.001 | <0.01 |
| RAPGEF3 | -2.66 | 8.83 | 457.53 | <0.001 | <0.01 |
| CD36 | -5.07 | 13.55 | 456.80 | <0.001 | <0.01 |
| FHL1 | -5.35 | 13.34 | 445.94 | <0.001 | <0.01 |
| CLEC3B | -4.34 | 9.54 | 422.89 | <0.001 | <0.01 |
| TUBB3 | 4.85 | 6.18 | 418.53 | <0.001 | <0.01 |
| GNG11 | -2.42 | 9.93 | 391.67 | <0.001 | <0.01 |
| ARRB1 | -1.62 | 9.65 | 389.39 | <0.001 | <0.01 |
| CABLES2 | 1.81 | 8.22 | 378.06 | <0.001 | <0.01 |
| PFN1 | 1.46 | 13.04 | 375.65 | <0.001 | <0.01 |
| CFL1 | 1.32 | 13.52 | 370.93 | <0.001 | <0.01 |
| MANF | 1.50 | 9.86 | 365.45 | <0.001 | <0.01 |
| KIF22 | 1.53 | 9.75 | 365.02 | <0.001 | <0.01 |
| MGLL | -2.83 | 11.69 | 355.07 | <0.001 | <0.01 |
| CFD | -4.25 | 11.03 | 332.88 | <0.001 | <0.01 |
| MMRN1 | -4.35 | 8.93 | 322.57 | <0.001 | <0.01 |
| KIF1A | 6.40 | 9.27 | 321.81 | <0.001 | <0.01 |
| VWF | -2.39 | 12.69 | 320.36 | <0.001 | <0.01 |
| F8 | -1.90 | 9.23 | 317.85 | <0.001 | <0.01 |
| CSK | 1.37 | 10.12 | 317.44 | <0.001 | <0.01 |
| EHD2 | -2.28 | 11.73 | 314.15 | <0.001 | <0.01 |
| SELENOP | -2.46 | 11.43 | 309.71 | <0.001 | <0.01 |
| HBB | -5.31 | 10.86 | 308.63 | <0.001 | <0.01 |
| ITPR1 | -2.45 | 10.58 | 306.53 | <0.001 | <0.01 |
| TAGLN2 | 1.48 | 13.53 | 297.52 | <0.001 | <0.01 |
| CYRIB | 1.18 | 10.36 | 290.97 | <0.001 | <0.01 |
| MMD | -2.80 | 10.18 | 286.86 | <0.001 | <0.01 |
| FN1 | 2.59 | 15.15 | 277.31 | <0.001 | <0.01 |
| H3C12 | 4.18 | 1.32 | 274.88 | <0.001 | <0.01 |
| KIF26B | 2.99 | 8.59 | 271.51 | <0.001 | <0.01 |
| YWHAZ | 1.19 | 13.97 | 268.31 | <0.001 | <0.01 |
| SRC | 1.35 | 9.99 | 263.44 | <0.001 | <0.01 |
| HDAC2 | 1.42 | 11.28 | 260.91 | <0.001 | <0.01 |
| H3C10 | 3.35 | 5.77 | 260.61 | <0.001 | <0.01 |
| CDK5 | 1.53 | 7.90 | 258.91 | <0.001 | <0.01 |
| PECAM1 | -1.76 | 12.29 | 249.67 | <0.001 | <0.01 |
| SYTL4 | -1.60 | 8.48 | 246.95 | <0.001 | <0.01 |
| TUBA1B | 1.08 | 11.99 | 246.30 | <0.001 | <0.01 |
| CALU | 1.33 | 12.21 | 245.27 | <0.001 | <0.01 |
| ADRA2A | -2.64 | 9.06 | 238.88 | <0.001 | <0.01 |
| GNG4 | 4.19 | 7.97 | 235.54 | <0.001 | <0.01 |
| P2RY12 | -2.92 | 6.01 | 231.18 | <0.001 | <0.01 |
| TTN | -5.81 | 12.22 | 223.86 | <0.001 | <0.01 |
| IGF1 | -2.85 | 7.13 | 222.06 | <0.001 | <0.01 |
| RGS10 | 1.60 | 9.41 | 221.41 | <0.001 | <0.01 |
| ABHD12 | 1.05 | 9.97 | 220.74 | <0.001 | <0.01 |
| TUBB4A | 4.27 | 5.93 | 203.19 | <0.001 | <0.01 |
| CDK2 | 1.06 | 9.25 | 201.39 | <0.001 | <0.01 |
| H3-3A | 1.16 | 9.54 | 201.28 | <0.001 | <0.01 |
| ORM2 | 5.94 | 6.61 | 199.93 | <0.001 | <0.01 |
| GNG5 | 1.13 | 10.35 | 196.89 | <0.001 | <0.01 |
| KIFC2 | 2.03 | 7.84 | 192.38 | <0.001 | <0.01 |
| RHOB | -1.63 | 12.41 | 188.02 | <0.001 | <0.01 |
| PIK3R1 | -1.56 | 11.97 | 187.81 | <0.001 | <0.01 |
| ALB | -5.16 | 9.28 | 187.20 | <0.001 | <0.01 |
| PROS1 | -1.62 | 9.72 | 186.93 | <0.001 | <0.01 |
| PRKAR2B | -2.62 | 11.39 | 184.44 | <0.001 | <0.01 |
| PPIA | 1.02 | 12.49 | 181.79 | <0.001 | <0.01 |
| KIF13B | -1.34 | 9.87 | 172.60 | <0.001 | <0.01 |
| ORM1 | 6.80 | 7.15 | 165.06 | <0.001 | <0.01 |
| KIF20B | 1.25 | 8.27 | 164.99 | <0.001 | <0.01 |
| KLC2 | 1.03 | 8.97 | 164.05 | <0.001 | <0.01 |
| TRPC6 | -1.48 | 6.73 | 160.18 | <0.001 | <0.01 |
| HBA1 | -4.59 | 3.11 | 158.90 | <0.001 | <0.01 |
| GNAI1 | -1.71 | 9.54 | 158.59 | <0.001 | <0.01 |
| TNFSF4 | 1.60 | 6.12 | 158.41 | <0.001 | <0.01 |
| H3C11 | 3.94 | -0.23 | 154.90 | <0.001 | <0.01 |
| MYLK | -1.84 | 12.49 | 154.71 | <0.001 | <0.01 |
| GNG12 | -1.15 | 11.17 | 154.66 | <0.001 | <0.01 |
| KIF3C | 1.13 | 8.93 | 154.44 | <0.001 | <0.01 |
| DOCK11 | -1.97 | 9.84 | 153.02 | <0.001 | <0.01 |
| GNB4 | 1.17 | 10.09 | 151.08 | <0.001 | <0.01 |
| H3C4 | 2.95 | 3.98 | 150.49 | <0.001 | <0.01 |
| RASGRP1 | 1.82 | 7.89 | 148.67 | <0.001 | <0.01 |
| H3C8 | 3.06 | 3.89 | 147.63 | <0.001 | <0.01 |
| GNG2 | -1.73 | 9.46 | 146.44 | <0.001 | <0.01 |
| SNCA | -1.56 | 6.39 | 146.24 | <0.001 | <0.01 |
| H3C7 | 3.00 | 0.73 | 143.29 | <0.001 | <0.01 |
| KIF4B | 2.30 | 0.34 | 143.17 | <0.001 | <0.01 |
| PTPN6 | 1.12 | 9.44 | 137.63 | <0.001 | <0.01 |
| PIK3R2 | 1.40 | 4.32 | 130.55 | <0.001 | <0.01 |
| VEGFA | 1.34 | 11.06 | 129.96 | <0.001 | <0.01 |
| ODC1 | 1.05 | 10.47 | 128.08 | <0.001 | <0.01 |
| GNAS | 1.12 | 14.69 | 126.82 | <0.001 | <0.01 |
| SELP | -2.06 | 8.07 | 124.57 | <0.001 | <0.01 |
| TUBB4B | 1.02 | 11.90 | 123.77 | <0.001 | <0.01 |
| LCK | 1.61 | 7.42 | 122.71 | <0.001 | <0.01 |
| TIMP1 | 1.41 | 12.04 | 119.94 | <0.001 | <0.01 |
| F13A1 | -2.05 | 10.31 | 119.66 | <0.001 | <0.01 |
| F5 | 2.41 | 6.40 | 116.09 | <0.001 | <0.01 |
| ITIH3 | -1.80 | 4.61 | 114.92 | <0.001 | <0.01 |
| ABHD6 | -1.21 | 7.44 | 114.66 | <0.001 | <0.01 |
| CCL5 | 1.63 | 9.49 | 113.76 | <0.001 | <0.01 |
| TUBB8 | 1.83 | 2.63 | 113.35 | <0.001 | <0.01 |
| DAPP1 | 1.57 | 8.08 | 112.66 | <0.001 | <0.01 |
| ACTN2 | -5.83 | 10.76 | 112.02 | <0.001 | <0.01 |
| IFNB1 | 3.68 | 0.07 | 105.92 | <0.001 | <0.01 |
| DOCK3 | 1.77 | 6.12 | 104.11 | <0.001 | <0.01 |
| RAC2 | 1.33 | 9.23 | 104.05 | <0.001 | <0.01 |
| ITPR3 | 1.22 | 10.57 | 103.36 | <0.001 | <0.01 |
| GNG13 | 3.63 | 1.56 | 101.38 | <0.001 | <0.01 |
| GNA15 | 1.30 | 7.88 | 98.20 | <0.001 | <0.01 |
| SERPING1 | -1.01 | 12.91 | 97.95 | <0.001 | <0.01 |
| SH2B2 | 1.46 | 6.60 | 95.46 | <0.001 | <0.01 |
| COL1A1 | 1.69 | 15.56 | 93.17 | <0.001 | <0.01 |
| IRF1 | 1.13 | 10.03 | 92.01 | <0.001 | <0.01 |
| H3C13 | 1.84 | 0.66 | 88.66 | <0.001 | <0.01 |
| KIF21A | 1.09 | 8.71 | 88.44 | <0.001 | <0.01 |
| FERMT3 | 1.19 | 8.61 | 86.83 | <0.001 | <0.01 |
| HBG2 | -2.53 | 1.63 | 86.68 | <0.001 | <0.01 |
| HGF | -1.42 | 7.12 | 83.97 | <0.001 | <0.01 |
| TIMP3 | -1.36 | 7.93 | 80.88 | <0.001 | <0.01 |
| ZFPM2 | -1.42 | 7.40 | 79.91 | <0.001 | <0.01 |
| H3C1 | 2.35 | 1.37 | 76.65 | <0.001 | <0.01 |
| APBB1IP | -1.09 | 8.79 | 71.35 | <0.001 | <0.01 |
| GATA5 | 3.65 | 3.44 | 70.01 | <0.001 | <0.01 |
| GNGT1 | 4.79 | 2.14 | 68.54 | <0.001 | <0.01 |
| RAB27B | -1.42 | 9.30 | 68.38 | <0.001 | <0.01 |
| ABCC4 | 1.11 | 7.84 | 67.63 | <0.001 | <0.01 |
| QSOX1 | 1.01 | 12.09 | 67.23 | <0.001 | <0.01 |
| AHSG | 2.72 | 0.19 | 65.42 | <0.001 | <0.01 |
| TUBA4A | 1.21 | 9.91 | 62.39 | <0.001 | <0.01 |
| GNG3 | 1.14 | 1.75 | 62.12 | <0.001 | <0.01 |
| H2BC21 | 1.09 | 9.03 | 61.57 | <0.001 | <0.01 |
| CXCL5 | 2.30 | 6.83 | 58.03 | <0.001 | <0.01 |
| GATA4 | 3.61 | 2.42 | 57.72 | <0.001 | <0.01 |
| GNG8 | 1.75 | 0.85 | 56.09 | <0.001 | <0.01 |
| IFNA10 | 3.12 | -0.61 | 52.61 | <0.001 | <0.01 |
| KIF12 | -1.54 | 7.78 | 51.11 | <0.001 | <0.01 |
| RARRES2 | -1.19 | 10.36 | 50.89 | <0.001 | <0.01 |
| KIF26A | -1.06 | 7.23 | 49.15 | <0.001 | <0.01 |
| TUBA4B | 1.87 | 1.33 | 48.85 | <0.001 | <0.01 |
| TUBB8B | 1.44 | 2.62 | 48.50 | <0.001 | <0.01 |
| F2 | 2.02 | 0.13 | 46.88 | <0.001 | <0.01 |
| ITGA2B | 1.03 | 4.17 | 46.12 | <0.001 | <0.01 |
| HRG | 2.75 | 0.07 | 45.52 | <0.001 | <0.01 |
| H3C6 | 1.14 | 4.02 | 44.92 | <0.001 | <0.01 |
| TUBAL3 | 1.39 | 4.29 | 42.96 | <0.001 | <0.01 |
| KNG1 | 2.80 | 0.48 | 42.12 | <0.001 | <0.01 |
| NFE2 | 1.36 | 4.01 | 42.06 | <0.001 | <0.01 |
| CTSW | 1.03 | 6.52 | 41.26 | <0.001 | <0.01 |
| H3C14 | 4.38 | 0.13 | 41.17 | <0.001 | <0.01 |
| SERPINA4 | -2.19 | 2.08 | 38.30 | <0.001 | <0.01 |
| DGKI | -1.00 | 6.53 | 36.18 | <0.001 | <0.01 |
| HBG1 | -2.53 | -0.76 | 35.32 | <0.001 | <0.01 |
| TUBA3E | -1.62 | 1.56 | 33.60 | <0.001 | <0.01 |
| GNAT3 | 4.19 | 2.32 | 32.17 | <0.001 | <0.01 |
| APOA1 | 1.08 | 1.37 | 31.53 | <0.001 | <0.01 |
| SCG3 | 1.67 | 4.68 | 31.26 | <0.001 | <0.01 |
| SERPINE1 | 1.04 | 9.84 | 31.24 | <0.001 | <0.01 |
| GATA3 | -1.08 | 10.93 | 30.18 | <0.001 | <0.01 |
| LEFTY2 | 1.66 | 5.87 | 29.33 | <0.001 | <0.01 |
| EGF | -1.44 | 8.29 | 29.26 | <0.001 | <0.01 |
| FGB | -2.44 | 4.34 | 28.05 | <0.001 | <0.01 |
| DGKB | -1.29 | 2.73 | 27.50 | <0.001 | <0.01 |
| IFNA4 | 1.61 | -0.86 | 27.36 | <0.001 | <0.01 |
| IFNA8 | -1.65 | -0.68 | 27.22 | <0.001 | <0.01 |
| TUBA3D | -1.05 | 3.56 | 27.07 | <0.001 | <0.01 |
| HBD | -1.58 | 0.72 | 26.59 | <0.001 | <0.01 |
| KIF19 | 1.12 | 5.39 | 24.24 | <0.001 | <0.01 |
| FGA | -2.87 | 2.86 | 24.09 | <0.001 | <0.01 |
| TUBA3C | 2.80 | 0.35 | 21.70 | <0.001 | <0.01 |
| PLG | 1.13 | -0.10 | 12.53 | <0.001 | <0.01 |
| TRPC7 | 1.31 | -0.62 | 10.04 | <0.01 | <0.01 |
| FGG | 1.54 | 5.27 | 9.28 | <0.01 | <0.01 |

**Supplementary Table 3.** Functional enrichment analyses based on the DEGs between TNBC and normal tissues.

**KEGG**

| ID | Description | GeneRatio | P. adj | Q. val | Count |
| --- | --- | --- | --- | --- | --- |
| hsa05034 | Alcoholism | 25/125 | <0.001 | <0.001 | 25 |
| hsa04926 | Relaxin signaling pathway | 20/125 | <0.001 | <0.001 | 20 |
| hsa04613 | Neutrophil extracellular trap formation | 23/125 | <0.001 | <0.001 | 23 |
| hsa04540 | Gap junction | 17/125 | <0.001 | <0.001 | 17 |
| hsa04611 | Platelet activation | 19/125 | <0.001 | <0.001 | 19 |
| hsa05163 | Human cytomegalovirus infection | 24/125 | <0.001 | <0.001 | 24 |
| hsa04151 | PI3K-Akt signaling pathway | 27/125 | <0.001 | <0.001 | 27 |
| hsa05170 | Human immunodeficiency virus 1 infection | 21/125 | <0.001 | <0.001 | 21 |
| hsa05167 | Kaposi sarcoma-associated herpesvirus infection | 20/125 | <0.001 | <0.001 | 20 |
| hsa04610 | Complement and coagulation cascades | 14/125 | <0.001 | <0.001 | 14 |
| hsa04725 | Cholinergic synapse | 15/125 | <0.001 | <0.001 | 15 |
| hsa04726 | Serotonergic synapse | 15/125 | <0.001 | <0.001 | 15 |
| hsa04713 | Circadian entrainment | 14/125 | <0.001 | <0.001 | 14 |
| hsa04062 | Chemokine signaling pathway | 18/125 | <0.001 | <0.001 | 18 |
| hsa05032 | Morphine addiction | 13/125 | <0.001 | <0.001 | 13 |
| hsa04728 | Dopaminergic synapse | 15/125 | <0.001 | <0.001 | 15 |
| hsa04724 | Glutamatergic synapse | 14/125 | <0.001 | <0.001 | 14 |
| hsa04014 | Ras signaling pathway | 19/125 | <0.001 | <0.001 | 19 |
| hsa04371 | Apelin signaling pathway | 15/125 | <0.001 | <0.001 | 15 |
| hsa05131 | Shigellosis | 19/125 | <0.001 | <0.001 | 19 |
| hsa04723 | Retrograde endocannabinoid signaling | 15/125 | <0.001 | <0.001 | 15 |
| hsa04727 | GABAergic synapse | 12/125 | <0.001 | <0.001 | 12 |
| hsa05020 | Prion disease | 18/125 | <0.001 | <0.001 | 18 |
| hsa05012 | Parkinson disease | 17/125 | <0.001 | <0.001 | 17 |
| hsa04015 | Rap1 signaling pathway | 15/125 | <0.001 | <0.001 | 15 |
| hsa04210 | Apoptosis | 12/125 | <0.001 | <0.001 | 12 |
| hsa05322 | Systemic lupus erythematosus | 12/125 | <0.001 | <0.001 | 12 |
| hsa05130 | Pathogenic Escherichia coli infection | 14/125 | <0.001 | <0.001 | 14 |
| hsa04510 | Focal adhesion | 14/125 | <0.001 | <0.001 | 14 |
| hsa04145 | Phagosome | 12/125 | <0.001 | <0.001 | 12 |
| hsa05202 | Transcriptional misregulation in cancer | 13/125 | <0.001 | <0.001 | 13 |
| hsa05171 | Coronavirus disease - COVID-19 | 14/125 | <0.001 | <0.001 | 14 |
| hsa05010 | Alzheimer disease | 18/125 | <0.001 | <0.001 | 18 |
| hsa05142 | Chagas disease | 9/125 | <0.001 | <0.001 | 9 |
| hsa05132 | Salmonella infection | 14/125 | <0.001 | <0.001 | 14 |
| hsa04810 | Regulation of actin cytoskeleton | 13/125 | <0.001 | <0.001 | 13 |
| hsa05165 | Human papillomavirus infection | 16/125 | <0.001 | <0.001 | 16 |
| hsa05205 | Proteoglycans in cancer | 12/125 | <0.001 | <0.001 | 12 |
| hsa05144 | Malaria | 6/125 | <0.001 | <0.001 | 6 |
| hsa05417 | Lipid and atherosclerosis | 12/125 | <0.001 | <0.001 | 12 |
| hsa04750 | Inflammatory mediator regulation of TRP channels | 8/125 | <0.001 | <0.001 | 8 |
| hsa04650 | Natural killer cell mediated cytotoxicity | 9/125 | <0.001 | <0.001 | 9 |
| hsa01521 | EGFR tyrosine kinase inhibitor resistance | 7/125 | <0.001 | <0.001 | 7 |
| hsa05016 | Huntington disease | 14/125 | <0.01 | <0.001 | 14 |
| hsa05160 | Hepatitis C | 9/125 | <0.01 | <0.01 | 9 |
| hsa04933 | AGE-RAGE signaling pathway in diabetic complications | 7/125 | <0.01 | <0.01 | 7 |
| hsa04630 | JAK-STAT signaling pathway | 9/125 | <0.01 | <0.01 | 9 |
| hsa05161 | Hepatitis B | 9/125 | <0.01 | <0.01 | 9 |
| hsa04620 | Toll-like receptor signaling pathway | 7/125 | <0.01 | <0.01 | 7 |
| hsa04530 | Tight junction | 9/125 | <0.01 | <0.01 | 9 |
| hsa04066 | HIF-1 signaling pathway | 7/125 | <0.01 | <0.01 | 7 |
| hsa05014 | Amyotrophic lateral sclerosis | 14/125 | <0.01 | <0.01 | 14 |
| hsa04668 | TNF signaling pathway | 7/125 | <0.01 | <0.01 | 7 |
| hsa04923 | Regulation of lipolysis in adipocytes | 5/125 | <0.01 | <0.01 | 5 |
| hsa04072 | Phospholipase D signaling pathway | 8/125 | <0.01 | <0.01 | 8 |
| hsa04370 | VEGF signaling pathway | 5/125 | <0.01 | <0.01 | 5 |
| hsa04730 | Long-term depression | 5/125 | <0.01 | <0.01 | 5 |
| hsa04935 | Growth hormone synthesis, secretion and action | 7/125 | <0.01 | <0.01 | 7 |
| hsa05143 | African trypanosomiasis | 4/125 | <0.01 | <0.01 | 4 |
| hsa05235 | PD-L1 expression and PD-1 checkpoint pathway in cancer | 6/125 | <0.01 | <0.01 | 6 |
| hsa05022 | Pathways of neurodegeneration - multiple diseases | 16/125 | <0.01 | <0.01 | 16 |
| hsa04623 | Cytosolic DNA-sensing pathway | 5/125 | <0.01 | <0.01 | 5 |
| hsa04929 | GnRH secretion | 5/125 | <0.01 | <0.01 | 5 |
| hsa04070 | Phosphatidylinositol signaling system | 6/125 | <0.05 | <0.01 | 6 |
| hsa05231 | Choline metabolism in cancer | 6/125 | <0.05 | <0.01 | 6 |
| hsa04022 | cGMP-PKG signaling pathway | 8/125 | <0.05 | <0.01 | 8 |
| hsa04914 | Progesterone-mediated oocyte maturation | 6/125 | <0.05 | <0.01 | 6 |
| hsa05146 | Amoebiasis | 6/125 | <0.05 | <0.01 | 6 |
| hsa05218 | Melanoma | 5/125 | <0.05 | <0.01 | 5 |
| hsa05164 | Influenza A | 8/125 | <0.05 | <0.01 | 8 |
| hsa05135 | Yersinia infection | 7/125 | <0.05 | <0.01 | 7 |
| hsa04625 | C-type lectin receptor signaling pathway | 6/125 | <0.05 | <0.01 | 6 |
| hsa04915 | Estrogen signaling pathway | 7/125 | <0.05 | <0.01 | 7 |
| hsa05162 | Measles | 7/125 | <0.05 | <0.01 | 7 |
| hsa05418 | Fluid shear stress and atherosclerosis | 7/125 | <0.05 | <0.01 | 7 |
| hsa04928 | Parathyroid hormone synthesis, secretion and action | 6/125 | <0.05 | <0.01 | 6 |
| hsa04971 | Gastric acid secretion | 5/125 | <0.05 | <0.01 | 5 |
| hsa05133 | Pertussis | 5/125 | <0.05 | <0.01 | 5 |
| hsa05212 | Pancreatic cancer | 5/125 | <0.05 | <0.01 | 5 |
| hsa04360 | Axon guidance | 8/125 | <0.05 | <0.01 | 8 |
| hsa04670 | Leukocyte transendothelial migration | 6/125 | <0.05 | <0.05 | 6 |
| hsa04662 | B cell receptor signaling pathway | 5/125 | <0.05 | <0.05 | 5 |
| hsa04218 | Cellular senescence | 7/125 | <0.05 | <0.05 | 7 |
| hsa04512 | ECM-receptor interaction | 5/125 | <0.05 | <0.05 | 5 |
| hsa04020 | Calcium signaling pathway | 9/125 | <0.05 | <0.05 | 9 |
| hsa05169 | Epstein-Barr virus infection | 8/125 | <0.05 | <0.05 | 8 |
| hsa05222 | Small cell lung cancer | 5/125 | <0.05 | <0.05 | 5 |
| hsa04213 | Longevity regulating pathway - multiple species | 4/125 | <0.05 | <0.05 | 4 |
| hsa05207 | Chemical carcinogenesis - receptor activation | 8/125 | <0.05 | <0.05 | 8 |
| hsa05215 | Prostate cancer | 5/125 | <0.05 | <0.05 | 5 |
| hsa01522 | Endocrine resistance | 5/125 | <0.05 | <0.05 | 5 |
| hsa04960 | Aldosterone-regulated sodium reabsorption | 3/125 | <0.05 | <0.05 | 3 |
| hsa04924 | Renin secretion | 4/125 | <0.05 | <0.05 | 4 |
| hsa05211 | Renal cell carcinoma | 4/125 | <0.05 | <0.05 | 4 |
| hsa04660 | T cell receptor signaling pathway | 5/125 | <0.05 | <0.05 | 5 |
| hsa04622 | RIG-I-like receptor signaling pathway | 4/125 | <0.05 | <0.05 | 4 |
| hsa04917 | Prolactin signaling pathway | 4/125 | <0.05 | <0.05 | 4 |

**GO (Biological processes)**

| ID | Description | GeneRatio | P. adj | Q. val | Count |
| --- | --- | --- | --- | --- | --- |
| GO:0007596 | blood coagulation | 69/177 | <0.001 | <0.001 | 69 |
| GO:0007599 | hemostasis | 69/177 | <0.001 | <0.001 | 69 |
| GO:0050817 | coagulation | 69/177 | <0.001 | <0.001 | 69 |
| GO:0002576 | platelet degranulation | 51/177 | <0.001 | <0.001 | 51 |
| GO:0030168 | platelet activation | 30/177 | <0.001 | <0.001 | 30 |
| GO:0072376 | protein activation cascade | 10/177 | <0.001 | <0.001 | 10 |
| GO:0072378 | blood coagulation, fibrin clot formation | 10/177 | <0.001 | <0.001 | 10 |
| GO:0000183 | rDNA heterochromatin assembly | 11/177 | <0.001 | <0.001 | 11 |
| GO:0007018 | microtubule-based movement | 24/177 | <0.001 | <0.001 | 24 |
| GO:0038111 | interleukin-7-mediated signaling pathway | 10/177 | <0.001 | <0.001 | 10 |
| GO:0030219 | megakaryocyte differentiation | 14/177 | <0.001 | <0.001 | 14 |
| GO:0042730 | fibrinolysis | 9/177 | <0.001 | <0.001 | 9 |
| GO:0045652 | regulation of megakaryocyte differentiation | 13/177 | <0.001 | <0.001 | 13 |
| GO:0006890 | retrograde vesicle-mediated transport, Golgi to endoplasmic reticulum | 13/177 | <0.001 | <0.001 | 13 |
| GO:0098760 | response to interleukin-7 | 10/177 | <0.001 | <0.001 | 10 |
| GO:0098761 | cellular response to interleukin-7 | 10/177 | <0.001 | <0.001 | 10 |
| GO:0019886 | antigen processing and presentation of exogenous peptide antigen via MHC class II | 13/177 | <0.001 | <0.001 | 13 |
| GO:0070828 | heterochromatin organization | 12/177 | <0.001 | <0.001 | 12 |
| GO:0070527 | platelet aggregation | 11/177 | <0.001 | <0.001 | 11 |
| GO:0002495 | antigen processing and presentation of peptide antigen via MHC class II | 13/177 | <0.001 | <0.001 | 13 |
| GO:0002504 | antigen processing and presentation of peptide or polysaccharide antigen via MHC class II | 13/177 | <0.001 | <0.001 | 13 |
| GO:0030195 | negative regulation of blood coagulation | 10/177 | <0.001 | <0.001 | 10 |
| GO:0034109 | homotypic cell-cell adhesion | 12/177 | <0.001 | <0.001 | 12 |
| GO:1900047 | negative regulation of hemostasis | 10/177 | <0.001 | <0.001 | 10 |
| GO:0030193 | regulation of blood coagulation | 11/177 | <0.001 | <0.001 | 11 |
| GO:1900046 | regulation of hemostasis | 11/177 | <0.001 | <0.001 | 11 |
| GO:0050819 | negative regulation of coagulation | 10/177 | <0.001 | <0.001 | 10 |
| GO:0031507 | heterochromatin assembly | 11/177 | <0.001 | <0.001 | 11 |
| GO:0050818 | regulation of coagulation | 11/177 | <0.001 | <0.001 | 11 |
| GO:0045785 | positive regulation of cell adhesion | 22/177 | <0.001 | <0.001 | 22 |
| GO:0061041 | regulation of wound healing | 13/177 | <0.001 | <0.001 | 13 |
| GO:0006335 | DNA replication-dependent nucleosome assembly | 8/177 | <0.001 | <0.001 | 8 |
| GO:0034723 | DNA replication-dependent nucleosome organization | 8/177 | <0.001 | <0.001 | 8 |
| GO:0022407 | regulation of cell-cell adhesion | 21/177 | <0.001 | <0.001 | 21 |
| GO:0031589 | cell-substrate adhesion | 19/177 | <0.001 | <0.001 | 19 |
| GO:0002478 | antigen processing and presentation of exogenous peptide antigen | 14/177 | <0.001 | <0.001 | 14 |
| GO:0061045 | negative regulation of wound healing | 10/177 | <0.001 | <0.001 | 10 |
| GO:0045814 | negative regulation of gene expression, epigenetic | 12/177 | <0.001 | <0.001 | 12 |
| GO:0019884 | antigen processing and presentation of exogenous antigen | 14/177 | <0.001 | <0.001 | 14 |
| GO:1903706 | regulation of hemopoiesis | 20/177 | <0.001 | <0.001 | 20 |
| GO:0048002 | antigen processing and presentation of peptide antigen | 14/177 | <0.001 | <0.001 | 14 |
| GO:1903034 | regulation of response to wounding | 13/177 | <0.001 | <0.001 | 13 |
| GO:0097549 | chromatin organization involved in negative regulation of transcription | 12/177 | <0.001 | <0.001 | 12 |
| GO:0060968 | regulation of gene silencing | 12/177 | <0.001 | <0.001 | 12 |
| GO:0006334 | nucleosome assembly | 12/177 | <0.001 | <0.001 | 12 |
| GO:0014068 | positive regulation of phosphatidylinositol 3-kinase signaling | 10/177 | <0.001 | <0.001 | 10 |
| GO:1903035 | negative regulation of response to wounding | 10/177 | <0.001 | <0.001 | 10 |
| GO:0048193 | Golgi vesicle transport | 18/177 | <0.001 | <0.001 | 18 |
| GO:0030099 | myeloid cell differentiation | 19/177 | <0.001 | <0.001 | 19 |
| GO:0034728 | nucleosome organization | 13/177 | <0.001 | <0.001 | 13 |
| GO:0060964 | regulation of gene silencing by miRNA | 11/177 | <0.001 | <0.001 | 11 |
| GO:0010810 | regulation of cell-substrate adhesion | 14/177 | <0.001 | <0.001 | 14 |
| GO:0140014 | mitotic nuclear division | 16/177 | <0.001 | <0.001 | 16 |
| GO:0045637 | regulation of myeloid cell differentiation | 15/177 | <0.001 | <0.001 | 15 |
| GO:0014065 | phosphatidylinositol 3-kinase signaling | 12/177 | <0.001 | <0.001 | 12 |
| GO:0034401 | chromatin organization involved in regulation of transcription | 12/177 | <0.001 | <0.001 | 12 |
| GO:0060147 | regulation of posttranscriptional gene silencing | 11/177 | <0.001 | <0.001 | 11 |
| GO:0060966 | regulation of gene silencing by RNA | 11/177 | <0.001 | <0.001 | 11 |
| GO:0007160 | cell-matrix adhesion | 14/177 | <0.001 | <0.001 | 14 |
| GO:0019882 | antigen processing and presentation | 14/177 | <0.001 | <0.001 | 14 |
| GO:0022409 | positive regulation of cell-cell adhesion | 15/177 | <0.001 | <0.001 | 15 |
| GO:0070371 | ERK1 and ERK2 cascade | 16/177 | <0.001 | <0.001 | 16 |
| GO:0010951 | negative regulation of endopeptidase activity | 14/177 | <0.001 | <0.001 | 14 |
| GO:0006338 | chromatin remodeling | 13/177 | <0.001 | <0.001 | 13 |
| GO:0042110 | T cell activation | 19/177 | <0.001 | <0.001 | 19 |
| GO:0010466 | negative regulation of peptidase activity | 14/177 | <0.001 | <0.001 | 14 |
| GO:0006333 | chromatin assembly or disassembly | 13/177 | <0.001 | <0.001 | 13 |
| GO:0045861 | negative regulation of proteolysis | 16/177 | <0.001 | <0.001 | 16 |
| GO:0015671 | oxygen transport | 5/177 | <0.001 | <0.001 | 5 |
| GO:0051222 | positive regulation of protein transport | 15/177 | <0.001 | <0.001 | 15 |
| GO:0048015 | phosphatidylinositol-mediated signaling | 12/177 | <0.001 | <0.001 | 12 |
| GO:0014066 | regulation of phosphatidylinositol 3-kinase signaling | 10/177 | <0.001 | <0.001 | 10 |
| GO:0031497 | chromatin assembly | 12/177 | <0.001 | <0.001 | 12 |
| GO:0048017 | inositol lipid-mediated signaling | 12/177 | <0.001 | <0.001 | 12 |
| GO:0007159 | leukocyte cell-cell adhesion | 16/177 | <0.001 | <0.001 | 16 |
| GO:0042744 | hydrogen peroxide catabolic process | 6/177 | <0.001 | <0.001 | 6 |
| GO:0018108 | peptidyl-tyrosine phosphorylation | 16/177 | <0.001 | <0.001 | 16 |
| GO:0000070 | mitotic sister chromatid segregation | 11/177 | <0.001 | <0.001 | 11 |
| GO:0071824 | protein-DNA complex subunit organization | 14/177 | <0.001 | <0.001 | 14 |
| GO:0006323 | DNA packaging | 13/177 | <0.001 | <0.001 | 13 |
| GO:0018212 | peptidyl-tyrosine modification | 16/177 | <0.001 | <0.001 | 16 |
| GO:0065004 | protein-DNA complex assembly | 13/177 | <0.001 | <0.001 | 13 |
| GO:1904951 | positive regulation of establishment of protein localization | 15/177 | <0.001 | <0.001 | 15 |
| GO:0007597 | blood coagulation, intrinsic pathway | 5/177 | <0.001 | <0.001 | 5 |
| GO:0040029 | regulation of gene expression, epigenetic | 12/177 | <0.001 | <0.001 | 12 |
| GO:0006959 | humoral immune response | 16/177 | <0.001 | <0.001 | 16 |
| GO:0048285 | organelle fission | 18/177 | <0.001 | <0.001 | 18 |
| GO:0000280 | nuclear division | 17/177 | <0.001 | <0.001 | 17 |
| GO:0042743 | hydrogen peroxide metabolic process | 7/177 | <0.001 | <0.001 | 7 |
| GO:0015669 | gas transport | 5/177 | <0.001 | <0.001 | 5 |
| GO:0070372 | regulation of ERK1 and ERK2 cascade | 14/177 | <0.001 | <0.001 | 14 |
| GO:0050730 | regulation of peptidyl-tyrosine phosphorylation | 13/177 | <0.001 | <0.001 | 13 |
| GO:0090303 | positive regulation of wound healing | 7/177 | <0.001 | <0.001 | 7 |
| GO:0052547 | regulation of peptidase activity | 17/177 | <0.001 | <0.001 | 17 |
| GO:0051918 | negative regulation of fibrinolysis | 4/177 | <0.001 | <0.001 | 4 |
| GO:0007052 | mitotic spindle organization | 9/177 | <0.001 | <0.001 | 9 |
| GO:0030194 | positive regulation of blood coagulation | 5/177 | <0.001 | <0.001 | 5 |
| GO:0031639 | plasminogen activation | 5/177 | <0.001 | <0.001 | 5 |
| GO:1900048 | positive regulation of hemostasis | 5/177 | <0.001 | <0.001 | 5 |
| GO:0050731 | positive regulation of peptidyl-tyrosine phosphorylation | 11/177 | <0.001 | <0.001 | 11 |
| GO:0090307 | mitotic spindle assembly | 7/177 | <0.001 | <0.001 | 7 |
| GO:0050820 | positive regulation of coagulation | 5/177 | <0.001 | <0.001 | 5 |
| GO:0052548 | regulation of endopeptidase activity | 16/177 | <0.001 | <0.001 | 16 |
| GO:0000819 | sister chromatid segregation | 11/177 | <0.001 | <0.001 | 11 |
| GO:0033622 | integrin activation | 5/177 | <0.001 | <0.001 | 5 |
| GO:0051255 | spindle midzone assembly | 4/177 | <0.001 | <0.001 | 4 |
| GO:0034114 | regulation of heterotypic cell-cell adhesion | 5/177 | <0.001 | <0.001 | 5 |
| GO:0007259 | receptor signaling pathway via JAK-STAT | 10/177 | <0.001 | <0.001 | 10 |
| GO:0000281 | mitotic cytokinesis | 7/177 | <0.001 | <0.001 | 7 |
| GO:0006953 | acute-phase response | 6/177 | <0.001 | <0.001 | 6 |
| GO:0007162 | negative regulation of cell adhesion | 13/177 | <0.001 | <0.001 | 13 |
| GO:0002687 | positive regulation of leukocyte migration | 9/177 | <0.001 | <0.001 | 9 |
| GO:0042501 | serine phosphorylation of STAT protein | 5/177 | <0.001 | <0.001 | 5 |
| GO:0046425 | regulation of receptor signaling pathway via JAK-STAT | 8/177 | <0.001 | <0.001 | 8 |
| GO:1903036 | positive regulation of response to wounding | 7/177 | <0.001 | <0.001 | 7 |
| GO:0033628 | regulation of cell adhesion mediated by integrin | 6/177 | <0.001 | <0.001 | 6 |
| GO:0032200 | telomere organization | 10/177 | <0.001 | <0.001 | 10 |
| GO:0097696 | receptor signaling pathway via STAT | 10/177 | <0.001 | <0.001 | 10 |
| GO:0051346 | negative regulation of hydrolase activity | 16/177 | <0.001 | <0.001 | 16 |
| GO:0051917 | regulation of fibrinolysis | 4/177 | <0.001 | <0.001 | 4 |
| GO:0002526 | acute inflammatory response | 8/177 | <0.001 | <0.001 | 8 |
| GO:0034446 | substrate adhesion-dependent cell spreading | 8/177 | <0.001 | <0.001 | 8 |
| GO:1902850 | microtubule cytoskeleton organization involved in mitosis | 9/177 | <0.001 | <0.001 | 9 |
| GO:0007051 | spindle organization | 10/177 | <0.001 | <0.001 | 10 |
| GO:1904892 | regulation of receptor signaling pathway via STAT | 8/177 | <0.001 | <0.001 | 8 |
| GO:0043491 | protein kinase B signaling | 12/177 | <0.001 | <0.001 | 12 |
| GO:0043406 | positive regulation of MAP kinase activity | 11/177 | <0.001 | <0.001 | 11 |
| GO:1903793 | positive regulation of anion transport | 16/177 | <0.001 | <0.001 | 16 |
| GO:0051225 | spindle assembly | 8/177 | <0.001 | <0.001 | 8 |
| GO:0034116 | positive regulation of heterotypic cell-cell adhesion | 4/177 | <0.001 | <0.001 | 4 |
| GO:0050708 | regulation of protein secretion | 12/177 | <0.001 | <0.001 | 12 |
| GO:0050863 | regulation of T cell activation | 13/177 | <0.001 | <0.001 | 13 |
| GO:1903039 | positive regulation of leukocyte cell-cell adhesion | 11/177 | <0.001 | <0.001 | 11 |
| GO:0008625 | extrinsic apoptotic signaling pathway via death domain receptors | 7/177 | <0.001 | <0.001 | 7 |
| GO:1903037 | regulation of leukocyte cell-cell adhesion | 13/177 | <0.001 | <0.001 | 13 |
| GO:0045921 | positive regulation of exocytosis | 7/177 | <0.001 | <0.001 | 7 |
| GO:0031295 | T cell costimulation | 6/177 | <0.001 | <0.001 | 6 |
| GO:2000352 | negative regulation of endothelial cell apoptotic process | 5/177 | <0.001 | <0.001 | 5 |
| GO:0031294 | lymphocyte costimulation | 6/177 | <0.001 | <0.001 | 6 |
| GO:1902041 | regulation of extrinsic apoptotic signaling pathway via death domain receptors | 6/177 | <0.001 | <0.001 | 6 |
| GO:0010811 | positive regulation of cell-substrate adhesion | 8/177 | <0.001 | <0.001 | 8 |
| GO:0032102 | negative regulation of response to external stimulus | 14/177 | <0.001 | <0.001 | 14 |
| GO:0051480 | regulation of cytosolic calcium ion concentration | 13/177 | <0.001 | <0.001 | 13 |
| GO:0071103 | DNA conformation change | 13/177 | <0.001 | <0.001 | 13 |
| GO:0033141 | positive regulation of peptidyl-serine phosphorylation of STAT protein | 4/177 | <0.001 | <0.001 | 4 |
| GO:0050870 | positive regulation of T cell activation | 10/177 | <0.001 | <0.001 | 10 |
| GO:0006816 | calcium ion transport | 14/177 | <0.001 | <0.001 | 14 |
| GO:0043405 | regulation of MAP kinase activity | 12/177 | <0.001 | <0.001 | 12 |
| GO:0002791 | regulation of peptide secretion | 12/177 | <0.001 | <0.001 | 12 |
| GO:0007204 | positive regulation of cytosolic calcium ion concentration | 12/177 | <0.001 | <0.001 | 12 |
| GO:0061640 | cytoskeleton-dependent cytokinesis | 7/177 | <0.001 | <0.001 | 7 |
| GO:0071902 | positive regulation of protein serine/threonine kinase activity | 12/177 | <0.001 | <0.001 | 12 |
| GO:1902042 | negative regulation of extrinsic apoptotic signaling pathway via death domain receptors | 5/177 | <0.001 | <0.001 | 5 |
| GO:0042542 | response to hydrogen peroxide | 8/177 | <0.001 | <0.001 | 8 |
| GO:0097529 | myeloid leukocyte migration | 10/177 | <0.001 | <0.001 | 10 |
| GO:0051897 | positive regulation of protein kinase B signaling | 9/177 | <0.001 | <0.001 | 9 |
| GO:0023061 | signal release | 15/177 | <0.001 | <0.001 | 15 |
| GO:0098869 | cellular oxidant detoxification | 7/177 | <0.01 | <0.001 | 7 |
| GO:0000302 | response to reactive oxygen species | 10/177 | <0.01 | <0.001 | 10 |
| GO:2001237 | negative regulation of extrinsic apoptotic signaling pathway | 7/177 | <0.01 | <0.001 | 7 |
| GO:0098813 | nuclear chromosome segregation | 11/177 | <0.01 | <0.001 | 11 |
| GO:0033139 | regulation of peptidyl-serine phosphorylation of STAT protein | 4/177 | <0.01 | <0.001 | 4 |
| GO:0051656 | establishment of organelle localization | 14/177 | <0.01 | <0.001 | 14 |
| GO:0033627 | cell adhesion mediated by integrin | 6/177 | <0.01 | <0.001 | 6 |
| GO:0033135 | regulation of peptidyl-serine phosphorylation | 8/177 | <0.01 | <0.001 | 8 |
| GO:0008277 | regulation of G protein-coupled receptor signaling pathway | 8/177 | <0.01 | <0.001 | 8 |
| GO:0033138 | positive regulation of peptidyl-serine phosphorylation | 7/177 | <0.01 | <0.001 | 7 |
| GO:0001819 | positive regulation of cytokine production | 14/177 | <0.01 | <0.001 | 14 |
| GO:0046651 | lymphocyte proliferation | 11/177 | <0.01 | <0.01 | 11 |
| GO:0006874 | cellular calcium ion homeostasis | 14/177 | <0.01 | <0.01 | 14 |
| GO:0006979 | response to oxidative stress | 14/177 | <0.01 | <0.01 | 14 |
| GO:0032943 | mononuclear cell proliferation | 11/177 | <0.01 | <0.01 | 11 |
| GO:0031032 | actomyosin structure organization | 9/177 | <0.01 | <0.01 | 9 |
| GO:0002286 | T cell activation involved in immune response | 7/177 | <0.01 | <0.01 | 7 |
| GO:0051924 | regulation of calcium ion transport | 10/177 | <0.01 | <0.01 | 10 |
| GO:0055074 | calcium ion homeostasis | 14/177 | <0.01 | <0.01 | 14 |
| GO:0007080 | mitotic metaphase plate congression | 5/177 | <0.01 | <0.01 | 5 |
| GO:1904036 | negative regulation of epithelial cell apoptotic process | 5/177 | <0.01 | <0.01 | 5 |
| GO:0017157 | regulation of exocytosis | 9/177 | <0.01 | <0.01 | 9 |
| GO:2001236 | regulation of extrinsic apoptotic signaling pathway | 8/177 | <0.01 | <0.01 | 8 |
| GO:1990748 | cellular detoxification | 7/177 | <0.01 | <0.01 | 7 |
| GO:2001233 | regulation of apoptotic signaling pathway | 12/177 | <0.01 | <0.01 | 12 |
| GO:0010959 | regulation of metal ion transport | 10/177 | <0.01 | <0.01 | 10 |
| GO:0051896 | regulation of protein kinase B signaling | 10/177 | <0.01 | <0.01 | 10 |
| GO:0032680 | regulation of tumor necrosis factor production | 8/177 | <0.01 | <0.01 | 8 |
| GO:0046879 | hormone secretion | 11/177 | <0.01 | <0.01 | 11 |
| GO:0002685 | regulation of leukocyte migration | 9/177 | <0.01 | <0.01 | 9 |
| GO:0070588 | calcium ion transmembrane transport | 11/177 | <0.01 | <0.01 | 11 |
| GO:0032640 | tumor necrosis factor production | 8/177 | <0.01 | <0.01 | 8 |
| GO:0022604 | regulation of cell morphogenesis | 11/177 | <0.01 | <0.01 | 11 |
| GO:0097237 | cellular response to toxic substance | 7/177 | <0.01 | <0.01 | 7 |
| GO:0043687 | post-translational protein modification | 12/177 | <0.01 | <0.01 | 12 |
| GO:1903555 | regulation of tumor necrosis factor superfamily cytokine production | 8/177 | <0.01 | <0.01 | 8 |
| GO:0090276 | regulation of peptide hormone secretion | 9/177 | <0.01 | <0.01 | 9 |
| GO:0070374 | positive regulation of ERK1 and ERK2 cascade | 9/177 | <0.01 | <0.01 | 9 |
| GO:0032760 | positive regulation of tumor necrosis factor production | 6/177 | <0.01 | <0.01 | 6 |
| GO:0018105 | peptidyl-serine phosphorylation | 11/177 | <0.01 | <0.01 | 11 |
| GO:0046883 | regulation of hormone secretion | 10/177 | <0.01 | <0.01 | 10 |
| GO:0072503 | cellular divalent inorganic cation homeostasis | 14/177 | <0.01 | <0.01 | 14 |
| GO:0009914 | hormone transport | 11/177 | <0.01 | <0.01 | 11 |
| GO:0070661 | leukocyte proliferation | 11/177 | <0.01 | <0.01 | 11 |
| GO:0071706 | tumor necrosis factor superfamily cytokine production | 8/177 | <0.01 | <0.01 | 8 |
| GO:0001818 | negative regulation of cytokine production | 12/177 | <0.01 | <0.01 | 12 |
| GO:0009306 | protein secretion | 12/177 | <0.01 | <0.01 | 12 |
| GO:0035592 | establishment of protein localization to extracellular region | 12/177 | <0.01 | <0.01 | 12 |
| GO:0002323 | natural killer cell activation involved in immune response | 4/177 | <0.01 | <0.01 | 4 |
| GO:1903557 | positive regulation of tumor necrosis factor superfamily cytokine production | 6/177 | <0.01 | <0.01 | 6 |
| GO:0030001 | metal ion transport | 14/177 | <0.01 | <0.01 | 14 |
| GO:0030865 | cortical cytoskeleton organization | 5/177 | <0.01 | <0.01 | 5 |
| GO:0031638 | zymogen activation | 5/177 | <0.01 | <0.01 | 5 |
| GO:2000351 | regulation of endothelial cell apoptotic process | 5/177 | <0.01 | <0.01 | 5 |
| GO:0097191 | extrinsic apoptotic signaling pathway | 9/177 | <0.01 | <0.01 | 9 |
| GO:0002690 | positive regulation of leukocyte chemotaxis | 6/177 | <0.01 | <0.01 | 6 |
| GO:0002551 | mast cell chemotaxis | 3/177 | <0.01 | <0.01 | 3 |
| GO:0050714 | positive regulation of protein secretion | 7/177 | <0.01 | <0.01 | 7 |
| GO:0071692 | protein localization to extracellular region | 12/177 | <0.01 | <0.01 | 12 |
| GO:0051702 | biological process involved in interaction with symbiont | 6/177 | <0.01 | <0.01 | 6 |
| GO:0032388 | positive regulation of intracellular transport | 9/177 | <0.01 | <0.01 | 9 |
| GO:0071900 | regulation of protein serine/threonine kinase activity | 14/177 | <0.01 | <0.01 | 14 |
| GO:0034113 | heterotypic cell-cell adhesion | 5/177 | <0.01 | <0.01 | 5 |
| GO:0061098 | positive regulation of protein tyrosine kinase activity | 5/177 | <0.01 | <0.01 | 5 |
| GO:1903532 | positive regulation of secretion by cell | 10/177 | <0.01 | <0.01 | 10 |
| GO:0072507 | divalent inorganic cation homeostasis | 14/177 | <0.01 | <0.01 | 14 |
| GO:0050920 | regulation of chemotaxis | 9/177 | <0.01 | <0.01 | 9 |
| GO:2001234 | negative regulation of apoptotic signaling pathway | 9/177 | <0.01 | <0.01 | 9 |
| GO:0060337 | type I interferon signaling pathway | 6/177 | <0.01 | <0.01 | 6 |
| GO:0046462 | monoacylglycerol metabolic process | 3/177 | <0.01 | <0.01 | 3 |
| GO:0097531 | mast cell migration | 3/177 | <0.01 | <0.01 | 3 |
| GO:0032147 | activation of protein kinase activity | 11/177 | <0.01 | <0.01 | 11 |
| GO:0071357 | cellular response to type I interferon | 6/177 | <0.01 | <0.01 | 6 |
| GO:0045807 | positive regulation of endocytosis | 6/177 | <0.01 | <0.01 | 6 |
| GO:0032148 | activation of protein kinase B activity | 4/177 | <0.01 | <0.01 | 4 |
| GO:0030183 | B cell differentiation | 7/177 | <0.01 | <0.01 | 7 |
| GO:0018209 | peptidyl-serine modification | 11/177 | <0.01 | <0.01 | 11 |
| GO:0098754 | detoxification | 7/177 | <0.01 | <0.01 | 7 |
| GO:1901653 | cellular response to peptide | 12/177 | <0.01 | <0.01 | 12 |
| GO:0051310 | metaphase plate congression | 5/177 | <0.01 | <0.01 | 5 |
| GO:0072577 | endothelial cell apoptotic process | 5/177 | <0.01 | <0.01 | 5 |
| GO:0050921 | positive regulation of chemotaxis | 7/177 | <0.01 | <0.01 | 7 |
| GO:0030198 | extracellular matrix organization | 12/177 | <0.01 | <0.01 | 12 |
| GO:0007059 | chromosome segregation | 11/177 | <0.01 | <0.01 | 11 |
| GO:0043062 | extracellular structure organization | 12/177 | <0.01 | <0.01 | 12 |
| GO:0045229 | external encapsulating structure organization | 12/177 | <0.01 | <0.01 | 12 |
| GO:0034340 | response to type I interferon | 6/177 | <0.01 | <0.01 | 6 |
| GO:0032715 | negative regulation of interleukin-6 production | 5/177 | <0.01 | <0.01 | 5 |
| GO:0019730 | antimicrobial humoral response | 7/177 | <0.01 | <0.01 | 7 |
| GO:0010812 | negative regulation of cell-substrate adhesion | 5/177 | <0.01 | <0.01 | 5 |
| GO:0002790 | peptide secretion | 12/177 | <0.01 | <0.01 | 12 |
| GO:0002285 | lymphocyte activation involved in immune response | 8/177 | <0.01 | <0.01 | 8 |
| GO:0007200 | phospholipase C-activating G protein-coupled receptor signaling pathway | 6/177 | <0.01 | <0.01 | 6 |
| GO:0002237 | response to molecule of bacterial origin | 11/177 | <0.01 | <0.01 | 11 |
| GO:0030038 | contractile actin filament bundle assembly | 6/177 | <0.01 | <0.01 | 6 |
| GO:0043149 | stress fiber assembly | 6/177 | <0.01 | <0.01 | 6 |
| GO:0062197 | cellular response to chemical stress | 11/177 | <0.01 | <0.01 | 11 |
| GO:0043550 | regulation of lipid kinase activity | 5/177 | <0.01 | <0.01 | 5 |
| GO:0032386 | regulation of intracellular transport | 11/177 | <0.01 | <0.01 | 11 |
| GO:0051047 | positive regulation of secretion | 10/177 | <0.01 | <0.01 | 10 |
| GO:0007229 | integrin-mediated signaling pathway | 6/177 | <0.01 | <0.01 | 6 |
| GO:0150115 | cell-substrate junction organization | 6/177 | <0.01 | <0.01 | 6 |
| GO:0033623 | regulation of integrin activation | 3/177 | <0.01 | <0.01 | 3 |
| GO:1903131 | mononuclear cell differentiation | 12/177 | <0.01 | <0.01 | 12 |
| GO:0003018 | vascular process in circulatory system | 9/177 | <0.01 | <0.01 | 9 |
| GO:0034599 | cellular response to oxidative stress | 10/177 | <0.01 | <0.01 | 10 |
| GO:0030072 | peptide hormone secretion | 9/177 | <0.01 | <0.01 | 9 |
| GO:0002793 | positive regulation of peptide secretion | 7/177 | <0.01 | <0.01 | 7 |
| GO:1900026 | positive regulation of substrate adhesion-dependent cell spreading | 4/177 | <0.01 | <0.01 | 4 |
| GO:0030098 | lymphocyte differentiation | 11/177 | <0.01 | <0.01 | 11 |
| GO:0009615 | response to virus | 11/177 | <0.01 | <0.01 | 11 |
| GO:0042493 | response to drug | 11/177 | <0.01 | <0.01 | 11 |
| GO:0060326 | cell chemotaxis | 10/177 | <0.01 | <0.01 | 10 |
| GO:0051209 | release of sequestered calcium ion into cytosol | 6/177 | <0.01 | <0.01 | 6 |
| GO:0051261 | protein depolymerization | 6/177 | <0.01 | <0.01 | 6 |
| GO:0019932 | second-messenger-mediated signaling | 10/177 | <0.01 | <0.01 | 10 |
| GO:0051283 | negative regulation of sequestering of calcium ion | 6/177 | <0.01 | <0.01 | 6 |
| GO:0050727 | regulation of inflammatory response | 11/177 | <0.01 | <0.01 | 11 |
| GO:0031109 | microtubule polymerization or depolymerization | 6/177 | <0.01 | <0.01 | 6 |
| GO:0032675 | regulation of interleukin-6 production | 7/177 | <0.01 | <0.01 | 7 |
| GO:1901983 | regulation of protein acetylation | 5/177 | <0.01 | <0.01 | 5 |
| GO:0051282 | regulation of sequestering of calcium ion | 6/177 | <0.01 | <0.01 | 6 |
| GO:0007019 | microtubule depolymerization | 4/177 | <0.01 | <0.01 | 4 |
| GO:0034614 | cellular response to reactive oxygen species | 7/177 | <0.01 | <0.01 | 7 |
| GO:0051057 | positive regulation of small GTPase mediated signal transduction | 5/177 | <0.01 | <0.01 | 5 |
| GO:0055013 | cardiac muscle cell development | 5/177 | <0.01 | <0.01 | 5 |
| GO:0051651 | maintenance of location in cell | 8/177 | <0.01 | <0.01 | 8 |
| GO:0003215 | cardiac right ventricle morphogenesis | 3/177 | <0.01 | <0.01 | 3 |
| GO:0007015 | actin filament organization | 12/177 | <0.01 | <0.01 | 12 |
| GO:0043434 | response to peptide hormone | 12/177 | <0.01 | <0.01 | 12 |
| GO:0032635 | interleukin-6 production | 7/177 | <0.01 | <0.01 | 7 |
| GO:0051928 | positive regulation of calcium ion transport | 6/177 | <0.01 | <0.01 | 6 |
| GO:0051303 | establishment of chromosome localization | 5/177 | <0.01 | <0.01 | 5 |
| GO:0051056 | regulation of small GTPase mediated signal transduction | 10/177 | <0.01 | <0.01 | 10 |
| GO:0051235 | maintenance of location | 10/177 | <0.01 | <0.01 | 10 |
| GO:0008406 | gonad development | 8/177 | <0.01 | <0.01 | 8 |
| GO:0002688 | regulation of leukocyte chemotaxis | 6/177 | <0.01 | <0.01 | 6 |
| GO:0051208 | sequestering of calcium ion | 6/177 | <0.01 | <0.01 | 6 |
| GO:0033630 | positive regulation of cell adhesion mediated by integrin | 3/177 | <0.05 | <0.01 | 3 |
| GO:0050000 | chromosome localization | 5/177 | <0.05 | <0.01 | 5 |
| GO:0045137 | development of primary sexual characteristics | 8/177 | <0.05 | <0.01 | 8 |
| GO:1903792 | negative regulation of anion transport | 8/177 | <0.05 | <0.01 | 8 |
| GO:0032496 | response to lipopolysaccharide | 10/177 | <0.05 | <0.01 | 10 |
| GO:0043330 | response to exogenous dsRNA | 4/177 | <0.05 | <0.01 | 4 |
| GO:0046461 | neutral lipid catabolic process | 4/177 | <0.05 | <0.01 | 4 |
| GO:0046464 | acylglycerol catabolic process | 4/177 | <0.05 | <0.01 | 4 |
| GO:0006898 | receptor-mediated endocytosis | 10/177 | <0.05 | <0.01 | 10 |
| GO:0060575 | intestinal epithelial cell differentiation | 3/177 | <0.05 | <0.01 | 3 |
| GO:2000114 | regulation of establishment of cell polarity | 3/177 | <0.05 | <0.01 | 3 |
| GO:0055006 | cardiac cell development | 5/177 | <0.05 | <0.01 | 5 |
| GO:0032970 | regulation of actin filament-based process | 11/177 | <0.05 | <0.01 | 11 |
| GO:0002718 | regulation of cytokine production involved in immune response | 5/177 | <0.05 | <0.01 | 5 |
| GO:0030101 | natural killer cell activation | 5/177 | <0.05 | <0.01 | 5 |
| GO:0001952 | regulation of cell-matrix adhesion | 6/177 | <0.05 | <0.01 | 6 |
| GO:0000910 | cytokinesis | 7/177 | <0.05 | <0.01 | 7 |
| GO:0051302 | regulation of cell division | 7/177 | <0.05 | <0.01 | 7 |
| GO:0032612 | interleukin-1 production | 6/177 | <0.05 | <0.01 | 6 |
| GO:0048260 | positive regulation of receptor-mediated endocytosis | 4/177 | <0.05 | <0.01 | 4 |
| GO:0051043 | regulation of membrane protein ectodomain proteolysis | 3/177 | <0.05 | <0.01 | 3 |
| GO:0051781 | positive regulation of cell division | 5/177 | <0.05 | <0.01 | 5 |
| GO:0045766 | positive regulation of angiogenesis | 7/177 | <0.05 | <0.01 | 7 |
| GO:1904018 | positive regulation of vasculature development | 7/177 | <0.05 | <0.01 | 7 |
| GO:0030595 | leukocyte chemotaxis | 8/177 | <0.05 | <0.05 | 8 |
| GO:0007265 | Ras protein signal transduction | 10/177 | <0.05 | <0.05 | 10 |
| GO:0072593 | reactive oxygen species metabolic process | 9/177 | <0.05 | <0.05 | 9 |
| GO:0015874 | norepinephrine transport | 3/177 | <0.05 | <0.05 | 3 |
| GO:1905523 | positive regulation of macrophage migration | 3/177 | <0.05 | <0.05 | 3 |
| GO:0002697 | regulation of immune effector process | 12/177 | <0.05 | <0.05 | 12 |
| GO:0097581 | lamellipodium organization | 5/177 | <0.05 | <0.05 | 5 |
| GO:0090316 | positive regulation of intracellular protein transport | 7/177 | <0.05 | <0.05 | 7 |
| GO:1902743 | regulation of lamellipodium organization | 4/177 | <0.05 | <0.05 | 4 |
| GO:0070301 | cellular response to hydrogen peroxide | 5/177 | <0.05 | <0.05 | 5 |
| GO:0002092 | positive regulation of receptor internalization | 3/177 | <0.05 | <0.05 | 3 |
| GO:0032878 | regulation of establishment or maintenance of cell polarity | 3/177 | <0.05 | <0.05 | 3 |
| GO:0043331 | response to dsRNA | 4/177 | <0.05 | <0.05 | 4 |
| GO:0097553 | calcium ion transmembrane import into cytosol | 6/177 | <0.05 | <0.05 | 6 |
| GO:0051953 | negative regulation of amine transport | 3/177 | <0.05 | <0.05 | 3 |
| GO:0006638 | neutral lipid metabolic process | 6/177 | <0.05 | <0.05 | 6 |
| GO:0006639 | acylglycerol metabolic process | 6/177 | <0.05 | <0.05 | 6 |
| GO:0050867 | positive regulation of cell activation | 11/177 | <0.05 | <0.05 | 11 |
| GO:0002367 | cytokine production involved in immune response | 5/177 | <0.05 | <0.05 | 5 |
| GO:0032956 | regulation of actin cytoskeleton organization | 10/177 | <0.05 | <0.05 | 10 |
| GO:0009636 | response to toxic substance | 8/177 | <0.05 | <0.05 | 8 |
| GO:1903829 | positive regulation of cellular protein localization | 9/177 | <0.05 | <0.05 | 9 |
| GO:0061097 | regulation of protein tyrosine kinase activity | 5/177 | <0.05 | <0.05 | 5 |
| GO:1904035 | regulation of epithelial cell apoptotic process | 5/177 | <0.05 | <0.05 | 5 |
| GO:0043551 | regulation of phosphatidylinositol 3-kinase activity | 4/177 | <0.05 | <0.05 | 4 |
| GO:1900024 | regulation of substrate adhesion-dependent cell spreading | 4/177 | <0.05 | <0.05 | 4 |
| GO:0051251 | positive regulation of lymphocyte activation | 10/177 | <0.05 | <0.05 | 10 |
| GO:0010469 | regulation of signaling receptor activity | 7/177 | <0.05 | <0.05 | 7 |
| GO:0060491 | regulation of cell projection assembly | 7/177 | <0.05 | <0.05 | 7 |
| GO:0022408 | negative regulation of cell-cell adhesion | 7/177 | <0.05 | <0.05 | 7 |
| GO:0003300 | cardiac muscle hypertrophy | 5/177 | <0.05 | <0.05 | 5 |
| GO:0042100 | B cell proliferation | 5/177 | <0.05 | <0.05 | 5 |
| GO:0048010 | vascular endothelial growth factor receptor signaling pathway | 5/177 | <0.05 | <0.05 | 5 |
| GO:0060191 | regulation of lipase activity | 5/177 | <0.05 | <0.05 | 5 |
| GO:0008286 | insulin receptor signaling pathway | 6/177 | <0.05 | <0.05 | 6 |
| GO:0014897 | striated muscle hypertrophy | 5/177 | <0.05 | <0.05 | 5 |
| GO:0033157 | regulation of intracellular protein transport | 8/177 | <0.05 | <0.05 | 8 |
| GO:0042098 | T cell proliferation | 7/177 | <0.05 | <0.05 | 7 |
| GO:0007044 | cell-substrate junction assembly | 5/177 | <0.05 | <0.05 | 5 |
| GO:0019229 | regulation of vasoconstriction | 4/177 | <0.05 | <0.05 | 4 |
| GO:1905330 | regulation of morphogenesis of an epithelium | 4/177 | <0.05 | <0.05 | 4 |
| GO:0014896 | muscle hypertrophy | 5/177 | <0.05 | <0.05 | 5 |
| GO:0002446 | neutrophil mediated immunity | 12/177 | <0.05 | <0.05 | 12 |
| GO:2000756 | regulation of peptidyl-lysine acetylation | 4/177 | <0.05 | <0.05 | 4 |
| GO:0002828 | regulation of type 2 immune response | 3/177 | <0.05 | <0.05 | 3 |
| GO:0150117 | positive regulation of cell-substrate junction organization | 3/177 | <0.05 | <0.05 | 3 |
| GO:0042119 | neutrophil activation | 12/177 | <0.05 | <0.05 | 12 |
| GO:0097530 | granulocyte migration | 6/177 | <0.05 | <0.05 | 6 |
| GO:0046822 | regulation of nucleocytoplasmic transport | 5/177 | <0.05 | <0.05 | 5 |
| GO:0071868 | cellular response to monoamine stimulus | 5/177 | <0.05 | <0.05 | 5 |
| GO:0071870 | cellular response to catecholamine stimulus | 5/177 | <0.05 | <0.05 | 5 |
| GO:0003180 | aortic valve morphogenesis | 3/177 | <0.05 | <0.05 | 3 |
| GO:0019835 | cytolysis | 3/177 | <0.05 | <0.05 | 3 |
| GO:0045907 | positive regulation of vasoconstriction | 3/177 | <0.05 | <0.05 | 3 |
| GO:0060402 | calcium ion transport into cytosol | 6/177 | <0.05 | <0.05 | 6 |
| GO:0043271 | negative regulation of ion transport | 9/177 | <0.05 | <0.05 | 9 |
| GO:0120034 | positive regulation of plasma membrane bounded cell projection assembly | 5/177 | <0.05 | <0.05 | 5 |
| GO:0038128 | ERBB2 signaling pathway | 3/177 | <0.05 | <0.05 | 3 |
| GO:0043254 | regulation of protein-containing complex assembly | 11/177 | <0.05 | <0.05 | 11 |
| GO:0000187 | activation of MAPK activity | 6/177 | <0.05 | <0.05 | 6 |
| GO:0051017 | actin filament bundle assembly | 6/177 | <0.05 | <0.05 | 6 |
| GO:0030100 | regulation of endocytosis | 7/177 | <0.05 | <0.05 | 7 |
| GO:0007548 | sex differentiation | 8/177 | <0.05 | <0.05 | 8 |
| GO:0050909 | sensory perception of taste | 4/177 | <0.05 | <0.05 | 4 |
| GO:0034764 | positive regulation of transmembrane transport | 7/177 | <0.05 | <0.05 | 7 |
| GO:0034767 | positive regulation of ion transmembrane transport | 7/177 | <0.05 | <0.05 | 7 |
| GO:0032611 | interleukin-1 beta production | 5/177 | <0.05 | <0.05 | 5 |
| GO:0071867 | response to monoamine | 5/177 | <0.05 | <0.05 | 5 |
| GO:0071869 | response to catecholamine | 5/177 | <0.05 | <0.05 | 5 |
| GO:0048738 | cardiac muscle tissue development | 7/177 | <0.05 | <0.05 | 7 |
| GO:0002724 | regulation of T cell cytokine production | 3/177 | <0.05 | <0.05 | 3 |
| GO:1905332 | positive regulation of morphogenesis of an epithelium | 3/177 | <0.05 | <0.05 | 3 |
| GO:0050918 | positive chemotaxis | 4/177 | <0.05 | <0.05 | 4 |
| GO:0002703 | regulation of leukocyte mediated immunity | 7/177 | <0.05 | <0.05 | 7 |
| GO:0071219 | cellular response to molecule of bacterial origin | 7/177 | <0.05 | <0.05 | 7 |
| GO:0071375 | cellular response to peptide hormone stimulus | 9/177 | <0.05 | <0.05 | 9 |
| GO:0002706 | regulation of lymphocyte mediated immunity | 6/177 | <0.05 | <0.05 | 6 |
| GO:0042113 | B cell activation | 9/177 | <0.05 | <0.05 | 9 |
| GO:0007191 | adenylate cyclase-activating dopamine receptor signaling pathway | 2/177 | <0.05 | <0.05 | 2 |
| GO:0035376 | sterol import | 2/177 | <0.05 | <0.05 | 2 |
| GO:0070508 | cholesterol import | 2/177 | <0.05 | <0.05 | 2 |
| GO:2000048 | negative regulation of cell-cell adhesion mediated by cadherin | 2/177 | <0.05 | <0.05 | 2 |
| GO:0061572 | actin filament bundle organization | 6/177 | <0.05 | <0.05 | 6 |
| GO:2000241 | regulation of reproductive process | 6/177 | <0.05 | <0.05 | 6 |
| GO:0010543 | regulation of platelet activation | 3/177 | <0.05 | <0.05 | 3 |
| GO:0007163 | establishment or maintenance of cell polarity | 7/177 | <0.05 | <0.05 | 7 |
| GO:0031623 | receptor internalization | 5/177 | <0.05 | <0.05 | 5 |
| GO:0150116 | regulation of cell-substrate junction organization | 4/177 | <0.05 | <0.05 | 4 |
| GO:0010517 | regulation of phospholipase activity | 4/177 | <0.05 | <0.05 | 4 |
| GO:0008608 | attachment of spindle microtubules to kinetochore | 3/177 | <0.05 | <0.05 | 3 |
| GO:0001909 | leukocyte mediated cytotoxicity | 5/177 | <0.05 | <0.05 | 5 |
| GO:0055007 | cardiac muscle cell differentiation | 5/177 | <0.05 | <0.05 | 5 |
| GO:0046579 | positive regulation of Ras protein signal transduction | 4/177 | <0.05 | <0.05 | 4 |
| GO:0032736 | positive regulation of interleukin-13 production | 2/177 | <0.05 | <0.05 | 2 |
| GO:0033625 | positive regulation of integrin activation | 2/177 | <0.05 | <0.05 | 2 |
| GO:0033632 | regulation of cell-cell adhesion mediated by integrin | 2/177 | <0.05 | <0.05 | 2 |
| GO:2000551 | regulation of T-helper 2 cell cytokine production | 2/177 | <0.05 | <0.05 | 2 |
| GO:0002369 | T cell cytokine production | 3/177 | <0.05 | <0.05 | 3 |
| GO:0003176 | aortic valve development | 3/177 | <0.05 | <0.05 | 3 |
| GO:0042092 | type 2 immune response | 3/177 | <0.05 | <0.05 | 3 |
| GO:0002696 | positive regulation of leukocyte activation | 10/177 | <0.05 | <0.05 | 10 |
| GO:0030032 | lamellipodium assembly | 4/177 | <0.05 | <0.05 | 4 |
| GO:0043154 | negative regulation of cysteine-type endopeptidase activity involved in apoptotic process | 4/177 | <0.05 | <0.05 | 4 |
| GO:0046834 | lipid phosphorylation | 4/177 | <0.05 | <0.05 | 4 |
| GO:1904019 | epithelial cell apoptotic process | 5/177 | <0.05 | <0.05 | 5 |
| GO:0016049 | cell growth | 11/177 | <0.05 | <0.05 | 11 |
| GO:0060193 | positive regulation of lipase activity | 4/177 | <0.05 | <0.05 | 4 |
| GO:0071542 | dopaminergic neuron differentiation | 3/177 | <0.05 | <0.05 | 3 |
| GO:0090218 | positive regulation of lipid kinase activity | 3/177 | <0.05 | <0.05 | 3 |
| GO:1902745 | positive regulation of lamellipodium organization | 3/177 | <0.05 | <0.05 | 3 |
| GO:0001558 | regulation of cell growth | 10/177 | <0.05 | <0.05 | 10 |
| GO:0042129 | regulation of T cell proliferation | 6/177 | <0.05 | <0.05 | 6 |
| GO:0043112 | receptor metabolic process | 6/177 | <0.05 | <0.05 | 6 |
| GO:0001667 | ameboidal-type cell migration | 11/177 | <0.05 | <0.05 | 11 |
| GO:0046503 | glycerolipid catabolic process | 4/177 | <0.05 | <0.05 | 4 |
| GO:0031346 | positive regulation of cell projection organization | 9/177 | <0.05 | <0.05 | 9 |
| GO:0032652 | regulation of interleukin-1 production | 5/177 | <0.05 | <0.05 | 5 |
| GO:0030866 | cortical actin cytoskeleton organization | 3/177 | <0.05 | <0.05 | 3 |
| GO:0032506 | cytokinetic process | 3/177 | <0.05 | <0.05 | 3 |
| GO:0042401 | cellular biogenic amine biosynthetic process | 3/177 | <0.05 | <0.05 | 3 |
| GO:0002819 | regulation of adaptive immune response | 6/177 | <0.05 | <0.05 | 6 |
| GO:0033629 | negative regulation of cell adhesion mediated by integrin | 2/177 | <0.05 | <0.05 | 2 |
| GO:2001135 | regulation of endocytic recycling | 2/177 | <0.05 | <0.05 | 2 |
| GO:0010639 | negative regulation of organelle organization | 9/177 | <0.05 | <0.05 | 9 |
| GO:0009791 | post-embryonic development | 4/177 | <0.05 | <0.05 | 4 |
| GO:0009309 | amine biosynthetic process | 3/177 | <0.05 | <0.05 | 3 |
| GO:0030212 | hyaluronan metabolic process | 3/177 | <0.05 | <0.05 | 3 |
| GO:0050892 | intestinal absorption | 3/177 | <0.05 | <0.05 | 3 |
| GO:1905521 | regulation of macrophage migration | 3/177 | <0.05 | <0.05 | 3 |
| GO:0060485 | mesenchyme development | 8/177 | <0.05 | <0.05 | 8 |
| GO:0060401 | cytosolic calcium ion transport | 6/177 | <0.05 | <0.05 | 6 |
| GO:0051224 | negative regulation of protein transport | 5/177 | <0.05 | <0.05 | 5 |
| GO:1990266 | neutrophil migration | 5/177 | <0.05 | <0.05 | 5 |
| GO:0007188 | adenylate cyclase-modulating G protein-coupled receptor signaling pathway | 7/177 | <0.05 | <0.05 | 7 |
| GO:0043312 | neutrophil degranulation | 11/177 | <0.05 | <0.05 | 11 |
| GO:0060284 | regulation of cell development | 11/177 | <0.05 | <0.05 | 11 |
| GO:0007212 | dopamine receptor signaling pathway | 3/177 | <0.05 | <0.05 | 3 |
| GO:0032467 | positive regulation of cytokinesis | 3/177 | <0.05 | <0.05 | 3 |
| GO:0032735 | positive regulation of interleukin-12 production | 3/177 | <0.05 | <0.05 | 3 |
| GO:0010634 | positive regulation of epithelial cell migration | 6/177 | <0.05 | <0.05 | 6 |
| GO:0048771 | tissue remodeling | 6/177 | <0.05 | <0.05 | 6 |
| GO:0002709 | regulation of T cell mediated immunity | 4/177 | <0.05 | <0.05 | 4 |
| GO:0010770 | positive regulation of cell morphogenesis involved in differentiation | 4/177 | <0.05 | <0.05 | 4 |
| GO:0032272 | negative regulation of protein polymerization | 4/177 | <0.05 | <0.05 | 4 |
| GO:0045913 | positive regulation of carbohydrate metabolic process | 4/177 | <0.05 | <0.05 | 4 |
| GO:0061844 | antimicrobial humoral immune response mediated by antimicrobial peptide | 4/177 | <0.05 | <0.05 | 4 |
| GO:0071621 | granulocyte chemotaxis | 5/177 | <0.05 | <0.05 | 5 |
| GO:0002921 | negative regulation of humoral immune response | 2/177 | <0.05 | <0.05 | 2 |
| GO:0010755 | regulation of plasminogen activation | 2/177 | <0.05 | <0.05 | 2 |
| GO:0031115 | negative regulation of microtubule polymerization | 2/177 | <0.05 | <0.05 | 2 |
| GO:0033604 | negative regulation of catecholamine secretion | 2/177 | <0.05 | <0.05 | 2 |
| GO:0035745 | T-helper 2 cell cytokine production | 2/177 | <0.05 | <0.05 | 2 |
| GO:0045741 | positive regulation of epidermal growth factor-activated receptor activity | 2/177 | <0.05 | <0.05 | 2 |
| GO:0002283 | neutrophil activation involved in immune response | 11/177 | <0.05 | <0.05 | 11 |
| GO:0042310 | vasoconstriction | 4/177 | <0.05 | <0.05 | 4 |
| GO:0002698 | negative regulation of immune effector process | 5/177 | <0.05 | <0.05 | 5 |
| GO:0071216 | cellular response to biotic stimulus | 7/177 | <0.05 | <0.05 | 7 |
| GO:0001953 | negative regulation of cell-matrix adhesion | 3/177 | <0.05 | <0.05 | 3 |
| GO:0010591 | regulation of lamellipodium assembly | 3/177 | <0.05 | <0.05 | 3 |
| GO:1904646 | cellular response to amyloid-beta | 3/177 | <0.05 | <0.05 | 3 |
| GO:1905314 | semi-lunar valve development | 3/177 | <0.05 | <0.05 | 3 |
| GO:0050796 | regulation of insulin secretion | 6/177 | <0.05 | <0.05 | 6 |
| GO:2000117 | negative regulation of cysteine-type endopeptidase activity | 4/177 | <0.05 | <0.05 | 4 |
| GO:2000243 | positive regulation of reproductive process | 4/177 | <0.05 | <0.05 | 4 |
| GO:0001906 | cell killing | 6/177 | <0.05 | <0.05 | 6 |
| GO:0035296 | regulation of tube diameter | 5/177 | <0.05 | <0.05 | 5 |
| GO:0097746 | blood vessel diameter maintenance | 5/177 | <0.05 | <0.05 | 5 |
| GO:1904950 | negative regulation of establishment of protein localization | 5/177 | <0.05 | <0.05 | 5 |
| GO:0090066 | regulation of anatomical structure size | 11/177 | <0.05 | <0.05 | 11 |
| GO:0014910 | regulation of smooth muscle cell migration | 4/177 | <0.05 | <0.05 | 4 |
| GO:0006509 | membrane protein ectodomain proteolysis | 3/177 | <0.05 | <0.05 | 3 |
| GO:0035150 | regulation of tube size | 5/177 | <0.05 | <0.05 | 5 |
| GO:0010975 | regulation of neuron projection development | 10/177 | <0.05 | <0.05 | 10 |
| GO:0043409 | negative regulation of MAPK cascade | 6/177 | <0.05 | <0.05 | 6 |
| GO:0038166 | angiotensin-activated signaling pathway | 2/177 | <0.05 | <0.05 | 2 |
| GO:0050930 | induction of positive chemotaxis | 2/177 | <0.05 | <0.05 | 2 |
| GO:1905049 | negative regulation of metallopeptidase activity | 2/177 | <0.05 | <0.05 | 2 |
| GO:0002065 | columnar/cuboidal epithelial cell differentiation | 4/177 | <0.05 | <0.05 | 4 |
| GO:2000273 | positive regulation of signaling receptor activity | 3/177 | <0.05 | <0.05 | 3 |
| GO:1900180 | regulation of protein localization to nucleus | 5/177 | <0.05 | <0.05 | 5 |
| GO:0010507 | negative regulation of autophagy | 4/177 | <0.05 | <0.05 | 4 |

**Supplementary Table 4.** GSEA based on the subgroups categorized by the risk signature.

| Pathway | Category | P. adj | NES | Gene number |
| --- | --- | --- | --- | --- |
| ECM RECEPTOR INTERACTION | KEGG | <0.001 | 2.34 | 83 |
| FOCAL ADHESION | KEGG | <0.001 | 2.30 | 199 |
| TGF BETA SIGNALING PATHWAY | KEGG | 0.012 | 2.05 | 85 |
| VASCULAR SMOOTH MUSCLE CONTRACTION | KEGG | 0.012 | 2.02 | 114 |
| VEGF_SIGNALING_PATHWAY | KEGG | 0.033 | 1.86 | 72 |
| BLOOD VESSEL ENDOTHELIAL CELL MIGRATION | GOBP | 0.001 | 2.17 | 158 |
| PLATELET DERIVED GROWTH FACTOR RECEPTOR SIGNALING PATHWAY | GOBP | 0.001 | 2.19 | 56 |
| REGULATION OF BLOOD PRESSURE | GOBP | 0.002 | 2.12 | 175 |
| REGULATION OF VASCULAR PERMEABILITY | GOBP | <0.001 | 2.29 | 42 |
| REGULATION OF VASCULATURE DEVELOPMENT | GOBP | <0.001 | 2.29 | 310 |
